# Towards resolving Lamiales relationships: insights from rapidly evolving chloroplast sequences

**DOI:** 10.1186/1471-2148-10-352

**Published:** 2010-11-12

**Authors:** Bastian Schäferhoff, Andreas Fleischmann, Eberhard Fischer, Dirk C Albach, Thomas Borsch, Günther Heubl, Kai F Müller

**Affiliations:** 1 Institute for Evolution and Biodiversity, University of Muenster, Hüfferstraße 1, 48149 Münster, Germany; 2 Department Biology, Systematic Botany and Mycology, Ludwig-Maximilians-Universität München, Menzinger Straße 67, D-80638 Munich, Germany; 3 Institut für Integrierte Naturwissenschaften - Biologie, Universität Koblenz-Landau, Universitätsstraße 1, 56070 Koblenz, Germany; 4 Institut für Biologie und Umweltwissenschaften (IBU), Carl von Ossietzky Universität Oldenburg, Carl von Ossietzky-Str. 9-11, 26111 Oldenburg, Germany; 5 Botanischer Garten und Botanisches Museum Berlin-Dahlem and Institute for Biology, Dahlem Center of Plant Sciences (DCPS), Freie Universität Berlin, Königin Luise-Straße 6-8, 14195 Berlin, Germany

## Abstract

**Background:**

In the large angiosperm order Lamiales, a diverse array of highly specialized life strategies such as carnivory, parasitism, epiphytism, and desiccation tolerance occur, and some lineages possess drastically accelerated DNA substitutional rates or miniaturized genomes. However, understanding the evolution of these phenomena in the order, and clarifying borders of and relationships among lamialean families, has been hindered by largely unresolved trees in the past.

**Results:**

Our analysis of the rapidly evolving *trnK/matK*, *trnL-F *and *rps16 *chloroplast regions enabled us to infer more precise phylogenetic hypotheses for the Lamiales. Relationships among the nine first-branching families in the Lamiales tree are now resolved with very strong support. Subsequent to Plocospermataceae, a clade consisting of Carlemanniaceae plus Oleaceae branches, followed by Tetrachondraceae and a newly inferred clade composed of Gesneriaceae plus Calceolariaceae, which is also supported by morphological characters. Plantaginaceae (incl. Gratioleae) and Scrophulariaceae are well separated in the backbone grade; Lamiaceae and Verbenaceae appear in distant clades, while the recently described Linderniaceae are confirmed to be monophyletic and in an isolated position.

**Conclusions:**

Confidence about deep nodes of the Lamiales tree is an important step towards understanding the evolutionary diversification of a major clade of flowering plants. The degree of resolution obtained here now provides a first opportunity to discuss the evolution of morphological and biochemical traits in Lamiales. The multiple independent evolution of the carnivorous syndrome, once in Lentibulariaceae and a second time in Byblidaceae, is strongly supported by all analyses and topological tests. The evolution of selected morphological characters such as flower symmetry is discussed. The addition of further sequence data from introns and spacers holds promise to eventually obtain a fully resolved plastid tree of Lamiales.

## Background

**Figure 1:**
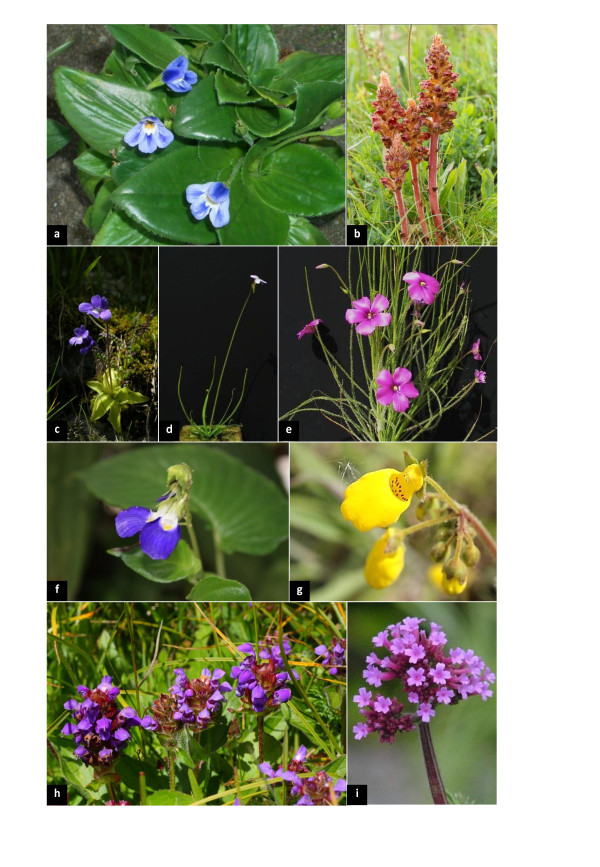
**Example taxa from Lamiales, showing representatives of desiccation-tolerant, parasitic, and carnivorous lineages, as well as members from families frequently referred to in the text**. **a**: the desiccation-tolerant *Craterostigma pumilum *from Linderniaceae; **b**: the holoparasitic *Orobanche gracilis *from Orobanchaceae, a family that contains all hemi- and holoparasites from Lamiales; **c**: *Pinguicula leptoceras *from Lentibulariaceae, the largest family of carnivorous plants in angiosperms; **d**: *Pinguicula filifolia*, with a habit resembling *Byblis; ***e**: *Byblis gigantea *from Byblidaceae, another carnivorous lineage previously suspected to be the closest relative of Lentibulariaceae; **f**: *Rhynchoglossum gardneri *from Gesneriaceae and **g ***Calceolaria andina *from Calceolariaceae, two families inferred here as sister groups based on molecular data, alveolated seeds and pair-flowered cymes; **h ***Prunella grandiflora *(Lamiaceae)**, i**: *Verbena bonariensis *(Verbenaceae)*; *both families were long regarded as close relatives but are inferred as only distantly related (Figure 2). Photos: a: E.F.; c, d, e: A.F.; f: Nadja Korotkova; g: D.C.A.; b, h, i: K.F.M.

With more than 23,000 species in at least 23 families [1], Lamiales (eudicots/asterids) are one of the largest orders of flowering plants, with representatives found all over the world. The highest diversity is contributed by herbaceous plants with mono-symmetric flowers. Some members are economically important, such as Lamiaceae (pot-herbs like mint, sage, oregano or basil), Oleaceae (olives), Pedaliaceae (sesame), Verbenaceae (timber, medicinal) Plantaginaceae (drugs like digitalis, ornamentals) and Scrophulariaceae (ornamentals). The order contains lineages with highly specialized life forms and traits of particular scientific interest. So far, their comparative study has been limited by the lack of a robust phylogenetic framework for Lamiales. Desiccation-tolerant members (so-called "resurrection plants", see Figure 1a) of the recently described family Linderniaceae [2] are a focus of molecular and evolutionary studies [3,2]. Extreme metabolic and genomic shifts are exhibited by parasitic plants. With Orobanchaceae, Lamiales harbor the largest number of parasitic angiosperms (Figure 1b). The family comprises both hemi- and holoparasites [4], with some species causing serious damage in agriculture [5]. Chloroplast genomes of members of Orobanchaceae show gene order rearrangements, high evolutionary rates and gene losses, potentially as a consequence of parasitism in this family. One line of current research in the family concentrates on gradual plastid evolution under increasingly relaxed functional constraints [Wicke et al., in prep].

### Carnivory in Lamiales

Lentibulariaceae, the most species-rich family of carnivorous plants (ca. 350 spp.) belongs to Lamiales (Figure 1c, d). This family is unique for a variety of reasons: traps of *Utricularia *(bladderworts) are regarded as a complex modification of leaves [6,7], and the typical angiosperm body plan is strongly relaxed in members of this genus [8-10]. *Utricularia *and its sister genus, *Genlisea *(the corkscrew plants), are the only carnivorous angiosperms known to feed on protozoa [11]. They have the smallest holoploid genome sizes among angiosperms, with some nuclear genomes as small as 63 Mbp or less [12], and exhibit the highest relative DNA substitution rates for some of the investigated chloroplast genome regions [13,14]. *Pinguicula *(butterworts), the third genus of Lentibulariaceae, is far less extreme in genome size, substitution rate and morphology, and exhibits glandular leaves that function as adhesive ("flypaper") traps (Figure 1c, d).

Apart from Lentibulariaceae, the monogeneric Australian family Byblidaceae (Figure 1e) also attracts and catches insects with simple flypaper traps comparable in function to those of *Pinguicula*. The carnivorous syndrome of *Byblis *was questioned by some authors, as the plants were considered to lack their own digestive enzymes and have not been demonstrated to be able to take up released nutrients, thus being ranked as merely "protocarnivorous" [15]. However, a recent study [16] detected phosphatase activity, thereby restoring the rank of carnivory to *Byblis*. Morphological links - flypaper trap leaves that are densely covered with multicellular, non-vascularized epidermal glands, as well as embryology [17,18] - and early phylogenetic studies suggested a sister relationship of Byblidaceae and Lentibulariaceae [19], thus hypothesizing a single origin of carnivory in the order, which was questioned later [14]. With the recently described genus *Philcoxia *[20], a further supposedly "protocarnivorous" lineage emerged and was placed in Lamiales [21]. Although a first test of enzymatic activity was negative [21], this might have been an artifact caused by the minuteness of the leaves, and further experiments to test its status as potentially fully carnivorous are underway.

Understanding the evolution of the morphological, ecological, and genomic peculiarities in the order heavily relies on having robust hypotheses on organismal relationships. For example, knowledge of the closest relatives of resurrection plants, parasites, and carnivores, respectively, would enable us to infer (pre-) adaptations and genomic changes on the evolutionary path leading to each of these specialized groups.

### Phylogeny and systematics of Lamiales: current state of knowledge

While the monophyly of many of the currently accepted families has been inferred with confidence by a number of molecular phylogenetic studies [22,23], there has been only little progress on understanding the relationships among families. Nearly all phylogenetic trees produced so far lacked resolution and support for inter-familiar relationships of Lamiales [24-26]. This has earned Lamiales the reputation of being among the most difficult angiosperm clades to resolve [27].

#### Circumscription of Lamiales and the inclusion of *Hydrostachys*

The current concept of Lamiales [28] expands the earlier order Lamiales from pre-cladistic classification systems [29,30] to also include former Scrophulariales and Oleales. While there is overwhelming evidence for the monophyly of Lamiales circumscribed like this [28], the surprising inclusion of *Hydrostachys *as an early branch in Lamiales was recently proposed [31]. *Hydrostachys *is a rheophyte from Africa and Madagascar suggested to be related to Cornales in most previous analyses of DNA sequence data, albeit without consistent placement in this order [32-34].

Most studies converged on a set of most likely candidates for the first branches of the Lamiales tree. Oleaceae have been consistently identified as being among the first branches [2,14,24,35]. Whenever the monotypic Plocospermataceae from Central America had been included in the sampling [26,35], they were found to be sister to the remaining Lamiales. In contrast, the Carlemanniaceae-suspected to have affinities of some kind to early branching Lamiales - have never been analyzed in the context of a broad Lamiales sampling. Tetrachondraceae have been resolved as a branch following Oleaceae [36,26].

#### No clear picture in more derived parts of tree

In contrast, there has not been any consistent hypothesis on the "backbone" of the remainder of the Lamiales tree [37,31]. Conflicting hypotheses have been put forward with regard to the relationships of Gesneriaceae and Calceolariaceae (Figure 1f, g) to each other and to remaining Lamiales. A successive branching order of Oleaceae, Calceolariaceae, Gesneriaceae, and remaining Lamiales was originally suggested [38,39], but support for the placement of Gesneriaceae and for the monophyly of the more derived remaining Lamiales was always negligible. On the other hand, a clade including Gesneriaceae and Calceolariaceae was hypothesized [2,40,41]. Consequently, relationships of Calceolariaceae remained indistinct, and until now there has been no study sampling all families from early branching Lamiales with a sufficient amount of sequence data to provide a clear picture.

The situation is even worse for the more derived, remaining lineages of the Lamiales tree - as far as the backbone and relationship among families is concerned, almost no resolution could be obtained by previous studies [42,31,43].

### The new circumscription of many traditional families

Lamiales are also known for the decomposition of previously widely accepted families due to phylogenetic insights.

#### Scrophulariaceae and Plantaginaceae

The most prominent case for a family that turned out to be polyphyletic are the Scrophulariaceae. In their traditional circumscription they used to be the largest family (more than 5000 spp. [44]) among Lamiales. In the first report on the polyphyly of Scrophulariaceae [45], members of the "old" Scrophulariaceae sensu lato were found in two different clades, named "scroph I" (including *Scrophularia*) and "scroph II" (containing *Plantago, Antirrhinum*, *Digitalis*, *Veronica*, *Hippuris *and *Callitriche*). The first clade was later [38] referred to as Scrophulariaceae sensu stricto (s. str.), while the "scroph II" clade was called Veronicaceae. However, since *Plantago *is contained in that clade, Plantaginaceae as the older name should be given priority and meanwhile became accepted for this clade [46,28]. Plantaginaceae experienced an enormous inflation since these early studies, when more and more genera from former Scrophulariaceae s. l. were included in phylogenetic studies and identified as members of this newly circumscribed family [22,37-39]. Some genera from tribe Gratioleae, including *Gratiola *itself, have been found in a well supported clade. Based on the unknown relationships to the the other lamialean families, it has been suggested to separate this part of the inflated Plantaginaceae by restoring family rank to former tribe Gratioleae from Scrophulariaceae as traditionally circumscribed [2].

#### Orobanchaceae

Initial molecular phylogenetic studies [47,48] showed that all hemi-parasitic members of the former Scrophulariaceae s. l. should be included in a newly circumscribed Orobanchaceae while the non-parasitic genus *Lindenbergia *was found sister to all hemi- and holoparasites and also included in Orobanchaceae. In this expanded circumscription [4,49], the monophyly of Orobanchaceae is strongly supported by all studies, and the family now comprises 89 genera with about 2000 species [49] and unites phototrophic, hemi- and holoparasitic plants. As next relatives to Orobanchaceae, a clade consisting of the East Asian genera *Rehmannia *(six species) and *Triaenophora *(one or two species) was identified recently [43,50].

#### Phrymaceae

Shortly after the first reports on the polyphyly of Scrophulariaceae [45], it was noticed that *Mimulus *(tribe Mimuleae) neither clustered with the "scroph I" nor the "scroph II" clade, but instead was found in a group together with Lamiaceae, *Paulownia *and Orobanchaceae [38]. Sampling the taxonomically isolated *Phryma *(Phrymaceae), but not *Mimulus*, *Phryma *appeared as sister to Orobanchaceae plus *Paulownia *[26]. In an attempt to redefine the Phrymaceae, their circumscription was expanded to include *Mimulus*, *Hemichaena*, *Berendtiella*, *Leucocarpus*, *Glossostigma*, *Peplidium*, *Elacholomia*, *Lancea*, and *Mazus *[51]. However, relationships to other families of Lamiales remained unclear. Sampling six genera from Phrymaceae [39], two clades emerged: one comprising *Mimulus*, *Phryma*, *Hemichaena *and *Berendita*, the other including *Mazus *and *Lancea *being sister to *Rehmannia*. Thus, the monophyly of Phrymaceae was put into question.

#### Linderniaceae

Linderniaceae were described as a new family independent from Scrophulariaceae, comprising genera formerly classified in the tribe Lindernieae of Scrophulariaceae s. l. and are characterized by stamens in which the abaxial filaments are conspicuously geniculate, zigzag shaped or spurred [2,52,53]. The original recognition as a distinct clade was based upon a taxon set including the genera *Artanema*, *Craterostigma*, *Crepidorhopalon*, *Torenia *and *Lindernia. *The existence of a Linderniaceae clade was confirmed by other studies comprising *Craterostigma*, *Lindernia*, *Torenia *and *Micranthemum *[22] or *Stemodiopsis*, *Micranthemum*, *Torenia *and *Picria *[39].

#### Calceolariaceae

*Jovellana *and *Calceolaria *(formerly Calceolarieae/Scrophulariaceae) were identified as another lineage separate from Scrophulariaceae, which led to recognizing them at family level (Calceolariaceae) [38]. The authors of this study initially also listed *Porodittia *as genus of this new family, but a subsequent study [41] showed *Porodittia *to be nested in *Calceolaria*.

#### Schlegeliaceae, Paulowniaceae, and Stilbaceae

The genera *Paulownia *and *Schlegelia*, which had been traditionally included either in Bignoniaceae or Scrophulariaceae, were not found to be related to any of these families based on molecular data [54] and therefore treated as families of their own [55,56]. In addition, *Halleria *was transferred from Scrophulariaceae to Stilbaceae [38]. Molecular phylogenetic studies later expanded the circumscription of Stilbaceae to a total of 11 genera [37,39].

### Aims of this study

Using a dataset representing all major lineages from Lamiales, the goal of the present study was to investigate inter-familial relationships within Lamiales, in the hope to come up with a better resolved tree that provides the basis for an interpretation of the evolution of the above-mentioned morphological, ecological, and molecular peculiarities observed in the order.

Since the protein-coding genes usually applied to the inference problem in Lamiales have not provided satisfactory resolution in the past, the approach in the current study was to employ non-coding and rapidly evolving chloroplast DNA. Introns and spacers have been demonstrated to be a valuable source of phylogenetic signal even on deeper taxonomic levels than they used to be applied to [57-59]. Mutational dynamics of non-coding regions also include microstructural changes in addition to substitutions, and generally are less constrained than coding genes [60]. Non-coding markers have been shown to be significantly more informative than coding regions [57]. Even more, non-coding markers have been successfully applied to disentangle deep nodes in angiosperm evolution [58].

## Methods

### Taxon sampling and plant material

Sequences from the plastid markers *trnK*/*matK*, *trnL-F *and *rps*16 were newly generated or downloaded from GenBank for 98 taxa from Lamiales, two outgroup taxa from Solanaceae, and one from Rubiaceae. All 23 families currently accepted for Lamiales [28] were sampled. Since one of the specific questions in our study was the relationship between Lentibulariaceae and Byblidaceae, which might have been blurred by long branch attraction (LBA) problems in previous studies, we slightly enhanced sampling for both families in one set of analyses and included two to three species for each genus. The complete material sampled is shown in Table 1. Using fewer representatives for either family did not change results. We also used a somewhat denser taxon sampling for Gratioleae (Plantaginaceae) in order to (i) examine whether the distinctness of this tribe [2] can be confirmed after taxan sampling enhancement and (ii) doublecheck the position of the apparently "protocarnivorous" genus *Philcoxia*.

**Table 1:** Taxa, specimens and GenBank acession numbers for sequences used in the present study

Genus	Family	*trnK/matK*	*trnLF*	*rps16*
***Acanthus***	Acanthaceae	*Acanthus longifolius *Poir.; [GenBank:AJ429326.1]	*Acanthus sennii *Chiov.; [GenBank:DQ054856.1]	*Acanthus sennii *Chiov.; [GenBank:DQ059148.1]
***Anastrabe***	Stilbaceae	*Anastrabe integerrima *E. Mey. Ex Benth.; H. Joffe 171; (M); [EMBL:FN773529]	*Anastrabe integerrima *E. Mey. Ex Benth.; H. Joffe 171; (M); [EMBL:FN794042 ]	*Anastrabe integerrima *E. Mey. Ex Benth.; [GenBank:AJ609216]
***Angelonia***	Plantaginaceae	*Angelonia sp*.; Löhne; BG Bonn; [EMBL:FN773530]	*Angelonia sp*.; Löhne; BG Bonn; [EMBL:FN794043]	*Angelonia sp*.; Löhne; BG Bonn; [EMBL:FN794079]
***Antirrhinum***	Plantaginaceae	*Antirrhinum majus *L.; [GenBank:AF051978]	*Antirrhinum majus *L.; [GenBank:AY316707]	*Antirrhinum majus *L.; [GenBank:AJ431054]
***Avicennia***	Acanthaceae	Avicennia germinans L.; [GenBank:AF531771]	Avicennia germinans L.; [GenBank:AY008819]	Avicennia marina (Forssk.) Vierh.; [GenBank:AJ431038]
***Bacopa***	Plantaginaceae	*Bacopa monnieri *(L.) Pennell; [GenBank:AY667458]	*Bacopa monnieri *(L.) Pennell; [GenBank:AY492170]	*Bacopa monnieri *(L.) Pennell; [GenBank:AY492196]
***Barthlottia***	Scrophulariaceae	*Barthlottia madagascariensis *Eb.Fisch.; A. Erpenbach s.n. (BONN); [EMBL:FN773531]	*Barthlottia madagascariensis *Eb.Fisch.; A. Erpenbach s.n. (BONN); [EMBL:FN794044]	*Barthlottia madagascariensis *Eb.Fisch.; A. Erpenbach s.n. (BONN); [EMBL:FN794080]
***Bryodes***	Linderniaceae	*Bryodes micrantha *Benth.; E. Fischer 10258; (BONN); [EMBL:FN773532]	*Bryodes micrantha *Benth.; E. Fischer 10258; Madagascar; (BONN); [EMBL:FN794045]	*Bryodes micrantha *Benth.; E. Fischer 10258; Madagascar; (BONN); [EMBL:FN794081]
***Buchnera***	Orobanchaceae	*Buchnera hispida *D. Don; E. Fischer 10230; (BONN); [EMBL:FN773533]	*Buchnera hispida *D. Don; E. Fischer 10230; (BONN); [EMBL:FN79046]	*Buchnera hispida *D. Don; E. Fischer 10230; (BONN); [EMBL:FN794082]
***Buddleja***	Scrophulariaceae	*Buddleja alternifolia *Maxim.; [GenBank:AF531772]	*Buddleja alternifolia *Maxim.; [GenBank:AF380857]	*Buddleja asiatica *Lour.; [GenBank:AJ431058]
***Byblis***	Byblidaceae	*Byblis gigantea *Lindl.; [GenBank:AF531774]	*Byblis gigantea *Lindl.; Kai Müller KM 733; (BONN); [EMBL:FN794047]	*Byblis gigantea *Lindl.; Kai Müller KM 733; (BONN); [EMBL:FN794083]
***Byblis***	Byblidaceae	*Byblis lamellata *Conran & Lowrie; Schäferhoff 49; (BONN); [EMBL:FN773534]	*Byblis lamellata *Conran & Lowrie; Schäferhoff 49; (BONN); [EMBL:FN794048]	*Byblis lamellata *Conrad & Lowrie; Schäferhoff 49; (BONN); [EMBL:FN794084]
***Byblis***	Byblidaceae	*Byblis liniflora *Salisb.; Schäferhoff 44; (BONN); [EMBL:FN773535]	*Byblis liniflora *Salisb.; Schäferhoff 44; (BONN); [EMBL:FN794049]	*Byblis liniflora *Salisb.; [GenBank:AJ431070]
***Calceolaria***	Calceolariaceae	*Calceolaria falklandica *Kraenzl.; [GenBank:AY667457.1]	*Calceolaria arachnoidea *Graham; [GenBank:AY423126]	*Calceolaria mexicana *Benth.; [GenBank:AJ609202]
***Callicarpa***	Lamiaceae	*Callicarpa bodinieri *H.Lév.; Schäferhoff 57; (BONN)	*Callicarpa japonica *Thunb.; [GenBank:AJ505536.1]	*Callicarpa japonica *Thunb.; [GenBank:AJ505413.1]
***Campsis***	Bignoniaceae	*Campsis radicans *Seem.; [GenBank:AF531775]	*Campsis radicans *Seem.; Kai Müller KM701; (BONN); [EMBL:FN794050]	*Campsis radicans *Seem.; Kai Müller KM701; (BONN); [EMBL:FN794085]
***Carlemannia***	Carlemanniaceae	*Carlemannia griffithii *Benth.; Grierson, A.J.C. & Long, D.D. 3027; (K); [EMBL:FN773536]	*Carlemannia griffithii *Benth.; Grierson, A.J.C. & Long, D.D. 3027; (K); [EMBL:FN794051]	*Carlemannia griffithii *Benth.; Grierson, A.J.C. & Long, D.D. 3027; (K); [EMBL:FN794086]
***Castilleja***	Orobanchaceae	*Castilleja linariifolia *Benth.; [GenBank:AF051981.1]	*Castilleja linariifolia *Benth.; [GenBank:EF103866.1]	*Castilleja integrifolia *L.f.; [GenBank:EF103789.1]
***Clerodendrum***	Lamiaceae	*Clerodendrum thomsoniae *Balf.; [GenBank:AY840129]	*Clerodendrum thomsoniae *Balf.; Schäferhoff 39; (BONN); [EMBL:FN794052]	*Clerodendrum thomsoniae *Balf.; Schäferhoff 39; (BONN); [EMBL:FN794087]
***Conobea***	Plantaginaceae	*Conobea multifida *(Michx.) Benth.; V. Mühlenbach 278; (M); [EMBL:FN773563]	*Conobea multifida *(Michx.) Benth.; V. Mühlenbach 278; (M); [EMBL:FN794053]	*Conobea multifida *(Michx.) Benth.; V. Mühlenbach 278; (M); [EMBL:FN794088]
***Craterostigma***	Linderniaceae	*Craterostigma hirsutum *S.Moore; [GenBank:AF531776]	*Craterostigma hirsutum *S.Moore; N. Peine 2; (BONN); [EMBL:FN794054]	*Craterostigma hirsutum *S.Moore; N. Peine 2; (BONN); [EMBL:FN794089]
***Dermatobotrys***	Scrophulariaceae	*Dermatobotrys saundersii *Bolus; B. Schäferhoff 64 (BONN); [EMBL:FN773537]	*Dermatobotrys saundersii *Bolus; [GenBank:AJ608596]	*Dermatobotrys saundersii *Bolus; [GenBank:AJ609191]
***Diascia***	Scrophulariaceae	*Diascia barbarae *Hook.f.; [GenBank:AY667464]	*Diascia capsularis *Benth.; [GenBank:AJ608595]	*Diascia capsularis *Benth.; [GenBank:AJ609190]
***Diclis***	Scrophulariaceae	*Diclis ovata *Benth.; E. Fischer 10255; (BONN); [EMBL:FN773538]	*Diclis ovata *Benth.; E. Fischer 10255; (BONN); [EMBL:FN794055]	*Diclis reptans *Benth.; [GenBank:AJ609188]
***Dipteracanthus***	Acanthaceae	*Dipteracanthus portellae *(Hook.f.) Boom; [GenBank:AF531773 ]	*Dipteracanthus portellae *(Hook.f.) Boom; Kai Müller KM734; (BONN); [EMBL:FN794056]	*Dipteracanthus portellae *(Hook.f.) Boom; Kai Müller KM734; (BONN); [EMBL:FN794090]
***Dodartia***	Phrymaceae	*Dodartia orientalis *L.; N. Hölzl M34434; (M); [EMBL:FN773539]	*Dodartia orientalis *L.; N. Hölzl M34434; (M); [EMBL:FN794057]	*Dodartia orientalis *L.; N. Hölzl M34434; (M); [EMBL:FN794091]
***Elytraria***	Acanthaceae	*Elytraria imbricata *(Vahl) Persoon; J. Calónico S.&A. Domínguez M. 4883; (M); [EMBL:FN773540]	*Elytraria imbricata *(Vahl) Persoon; [GenBank:AF061819.1]	*Elytraria imbricata *(Vahl) Persoon; P. Döbbeler 4189; (M); [EMBL:FN794092]
***Euphrasia***	Orobanchaceae	*Euphrasia stricta *D. Wolff ex J.F. Lehmann; Borsch 3785; (BONN); [EMBL:FN773541]	*Euphrasia stricta *D. Wolff ex J.F. Lehmann; Borsch 3785; (BONN); [EMBL:FN794058]	*Euphrasia stricta *D. Wolff ex J.F. Lehmann; Borsch 3785; (BONN); [EMBL:FN794093]
***Forsythia***	Oleaceae	*Forsythia suspensa *Vahl; [GenBank:EU281175.1]	*Forsythia suspensa *Vahl; [GenBank:EU281157.1]	*Forsythia suspensa *Vahl; [GenBank:AF225231.1]
***Genlisea***	Lentibulariaceae	*Genlisea aurea *A.St.-Hil.; [GenBank:AF531814.1]	*Genlisea aurea *A.St.-Hil.; [GenBank:AF482614]	*Genlisea aurea *A.St.-Hil.; [GenBank:AF482540]
***Genlisea***	Lentibulariaceae	*Genlisea hispidula *Stapf; [GenBank:AF531815]	*Genlisea hispidula *Stapf; [GenBank:AF488528.1]	*Genlisea hispidula *Stapf; [GenBank:AF488523.1]
***Globularia***	Plantaginaceae	*Globularia nudicaulis *L.; [GenBank:AY667473]	*Globularia trichosantha *Fisch. & C.A.Mey.; [GenBank:AY591321]	*Globularia repens *Lam.; [GenBank:AY492206]
***Gratiola***	Plantaginaceae	*Gratiola officinalis *L.; [GenBank:AF531777]	*Gratiola brevifolia *Raf.; [GenBank:AY727201 and AY727237]	*Gratiola pilosa *Michx.; [GenBank:AJ609182]
***Halleria***	Stilbaceae	*Halleria tetragona *Baker; [GenBank:AY667476.1]	*Halleria elliptica *L.; [GenBank:AJ621108]	*Halleria lucida *L.; [GenBank:AJ609181]
***Harpagophytum***	Pedaliaceae	*Harpagophytum grandidieri *Baill.; [GenBank:AF531813]	*Harpagophytum grandidieri *Baill.; [GenBank:AF482610]	*Harpagophytum grandidieri *Baill.; Kai Müller KM707; (BONN); [EMBL:FN794094]
***Harveya***	Orobanchaceae	*Harveya alba *Hepper; E. Fischer 11547; (BONN); [EMBL:FN773564]	*Harveya alba *Hepper; E. Fischer 11547; (BONN); [EMBL:FN794078]	*Harveya alba *Hepper; E. Fischer 11547; (BONN); [EMBL:FN794095]
***Hydrotriche***	Plantaginaceae	*Hydrotriche hottoniaeflora *Zucc.; E. Fischer 10264; (BONN); [EMBL:FN773542]	*Hydrotriche hottoniaeflora *Zucc.; E. Fischer 10264; (BONN); [EMBL:FN794059]	*Hydrotriche hottoniaeflora *Zucc.; E. Fischer 10264; (BONN); [EMBL:FN794096]
***Ibicella***	Martyniaceae	*Ibicella lutea *v.Eselt; [GenBank:AF531778]	*Ibicella lutea *v.Eselt; Kai Müller KM735; (BONN); [EMBL:FN794060]	*Ibicella lutea *v.Eselt; Kai Müller KM735; (BONN); [EMBL:FN794097]
***Jacaranda***	Bignoniaceae	*Jacaranda mimosifolia *D.Don; [GenBank:AJ429328.1]	*Jacaranda mimosifolia *D.Don; [GenBank:EF105070.1]	*Jacaranda mimosifolia *D.Don; [GenBank:AJ431039.1]
***Jasminum***	Oleaceae	*Jasminum nudiflorum *Lindl.; [GenBank:AF531779.1]	*Jasminum nudiflorum *Lindl.; [GenBank:AF531779.1]	*Jasminum nudiflorum *Lindl.; [GenBank:AF531779.1]
***Jovellana***	Calceolariaceae	*Jovellana violacea *G.Don; [GenBank:AJ580487.1]	*Jovellana violacea *G.Don; K.H. & W. Rechinger 63014; (M); [EMBL:FN794061]	*Jovellana violacea *G.Don; K.H. & W. Rechinger 63014; (M); [EMBL:FN794098]
***Kigelia***	Bignoniaceae	*Kigelia africana *Benth.; [GenBank:AF051988]	*Kigelia africana *Benth.; [GenBank:EF105072]	-
***Kohleria***	Gesneriaceae	*Kohleria spicata *Oerst.; [GenBank:AJ580486.1]	*Kohleria spicata *Oerst.; [GenBank:AJ439820.1]	*Kohleria ocellata *Fritsch in Engl. & Prantl; B. Schäferhoff 70; (BONN); [EMBL:FN794099]
***Lamium***	Lamiaceae	*Lamium maculatum *L.; [GenBank:AF531780]	*Lamium amplexicaule *L.; [GenBank:AB266235]	*Lamium album *L.; [GenBank:AJ431044]
***Lantana***	Verbenaceae	*Lantana camara *L.; [GenBank:AF315303.1]	*Lantana camara *L.; [GenBank:AF231884.1]	*Lantana camara *L.; [GenBank:EU348856.1]
***Limnophila***	Plantaginaceae	*Limnophila aromatica *(Lam.) Merr.; Schäferhoff 52; (BONN); [EMBL:FN773543]	*Limnophila aromatica *(Lam.) Merr.; Schäferhoff 52; (BONN); [EMBL:FN794062]	*Limnophila aromatica *(Lam.) Merr.; Schäferhoff 52; (BONN); [EMBL:FN794100]
***Limosella***	Scrophulariaceae	*Limosella aquatica *L.; Kai Müller & Andreas Worberg 258; (BONN); [EMBL:FN773544]	*Limosella aquatica *L.; Kai Müller & Andreas Worberg258; (BONN); [EMBL:FN794063]	*Limosella grandiflora *Benth.; [GenBank:AJ609170]
***Lindenbergia***	Orobanchaceae	*Lindenbergia philippinensis *Benth.; [GenBank:AF051990]	*Lindenbergia philippinensis *Benth.; [GenBank:AJ608586.1]	*Lindenbergia sp*.; [GenBank:AJ431049]
***Lindernia***	Linderniaceae	*Lindernia brevidens *Skan; E. Fischer 8022; (BONN); [EMBL:FN773545]	*Lindernia brevidens *Skan; [GenBank:AY492182]	*Lindernia brevidens *Skan; [GenBank:AY492213]
***Littorella***	Plantaginaceae	*Littorella uniflora *(L.) Asch.; N. Korotkova, K. Lewejohann & W. Lobin 2; (BONN); [EMBL:FN773546]	*Littorella uniflora *(L.) Asch.; N. Korotkova, K. Lewejohann & W. Lobin 2; (BONN); [EMBL:FN794064]	*Littorella uniflora *(L.) Asch.; N. Korotkova, K. Lewejohann & W. Lobin 2; (BONN); [EMBL:FN794101]
***Mazus***	Phrymaceae	*Mazus rugosus *Lour.; E. Fischer 10250; (BONN); [EMBL:FN773547]	*Mazus rugosus *Lour.; E. Fischer 10250; (BONN); [EMBL:FN794065]	*Mazus stachydifolius *Maxim.; AJ609167
***Mecardonia***	Plantaginaceae	*Mecardonia procumbens *Small; [GenBank:AY492152.1]	*Mecardonia procumbens *Small; [GenBank:AY492184]	*Mecardonia procumbens *Small; [GenBank:AY492215]
***Micranthemum***	Linderniaceae	*Micranthemum umbrosum *(J.F.Gmel.) Blake; Schäferhoff 43; (BONN); [EMBL:FN773548]	*Micranthemum umbrosum *(J.F.Gmel.) Blake; [GenBank:AY492186]	*Micranthemum umbrosum *(J.F.Gmel.) Blake; [GenBank:AY492217]
***Micrargeria***	Orobanchaceae	*Micrargeria filiformis *(Schum. Thonn.) Hutch. Dalziel; E. Fischer 10299; (BONN); [EMBL:FN773549]	*Micrargeria filiformis *(Schum. Thonn.) Hutch. Dalziel; E. Fischer 10299; (BONN); [EMBL:FN794066]	*Micrargeria filiformis *(Schum. Thonn.) Hutch. Dalziel; E. Fischer 10299; (BONN); [EMBL:FN794102]
***Mimulus***	Phrymaceae	*Mimulus guttatus *D.C.; [GenBank:AY667471]	*Mimulus micranthus *A. Heller; [GenBank:AY575534]	*Mimulus aurantiacus *Curtis; [GenBank:AJ609163]
***Mitraria***	Gesneriaceae	*Mitraria coccinea *Cav.; B. Schäferhoff 65; (BONN); [EMBL:FN773550]	*Mitraria coccinea *Cav.; B. Schäferhoff 65; (BONN); [EMBL:FN794067]	*Mitraria coccinea *Cav.; B. Schäferhoff 65; (BONN); [EMBL:FN794103]
***Myoporum***	Scrophulariaceae	*Myoporum montanum *R.Br.; [GenBank:AF531808]	*Myoporum montanum *R.Br.; [GenBank:AJ296513]	*Myoporum mauritianum *A.DC.; [GenBank:AJ609161]
***Ocimum***	Lamiaceae	*Ocimum basilicum *L.; [GenBank:AY177670.1]	*Ocimum basilicum *L.; [GenBank:AY570462.1]	*Ocimum basilicum *L.; [GenBank:AJ505351.1]
***Oftia***	Scrophulariaceae	*Oftia africana *Bocq. Ex Baill.; Schäferhoff 66.; (BONN); [EMBL:FN773551]	*Oftia africana *Bocq. Ex Baill.; Schäferhoff 66.; (BONN); [EMBL:FN794068]	*Oftia africana *Bocq. Ex Baill.; [GenBank:AJ609156.1]
***Olea***	Oleaceae	*Olea europaea *L.; [GenBank:AM229542.1]	*Olea europaea *L.; [GenBank:AM229542.1]	*Olea europaea *L.; [GenBank:AM229542.1]
***Orobanche***	Orobanchaceae	*Orobanche caryophyllacea *Sm.; [GenBank:AF051992]	*Orobanche coerulescens *Stephan; [GenBank:AY881137]	*Orobanche hederae *Duby; [GenBank:AJ431050]
***Otacanthus***	Plantaginaceae	*Otacanthus coeruleus *Lindl.; [GenBank:AY667459]	*Otacanthus sp*.; [GenBank:AY492188]	*Otacanthus sp*.; [GenBank:AY492219]
***Paulownia***	Paulowniaceae	*Paulownia tomentosa *(Thunb.) Steud.; [GenBank:AF051997]	*Paulownia tomentosa *(Thunb.) Steud.; [GenBank:AY423122]	*Paulownia tomentosa *(Thunb.) Steud.; [GenBank:AJ431051]
***Pedicularis***	Orobanchaceae	*Pedicularis sylvatica *L.; [GenBank:AF531781]	*Pedicularis cheilanthifolia *Schrenk; [GenBank:AY881133]	*Pedicularis attollens *A. Gray; [GenBank:EF103821]
***Petrea***	Verbenaceae	*Petrea racemosa *Nees; Schäferhoff 55; BG Bonn 11113; (BONN); [EMBL:FN773552]	*Petrea racemosa *Nees; Schäferhoff 55; BG Bonn 11113; (BONN); [EMBL:FN794069]	*Petrea racemosa *Nees; Schäferhoff 55; BG Bonn 11113; (BONN); [EMBL:FN794104]
***Philcoxia***	Plantaginaceae	*Philcoxia minensis *V.C.Souza & Giul.; [GenBank:EF467901]	*Philcoxia minensis *V.C.Souza & Giul.; [GenBank:EF467889.1]	-
***Phryma***	Phrymaceae	*Phryma leptostachya *L.; [GenBank:AJ429341.1]	*Phryma leptostachya *L.; [GenBank:DQ532471.1]	*Phryma leptostachya *L.; [GenBank:AJ431053.1]
***Phyla***	Verbenaceae	*Phyla nodiflora *(L.) Greene; Schäferhoff 56; BG Bonn 4146; (BONN); [EMBL:FN773553]	*Phyla nodiflora *(L.) Greene; Schäferhoff 56; BG Bonn 4146; (BONN); [EMBL:794070]	*Phyla nodiflora *(L.) Greene; Schäferhoff 56; BG Bonn 4146; (BONN); [EMBL:FN794105]
***Pinguicula***	Lentibulariaceae	*Pinguicula agnata *Casper; [GenBank:AF531782]	*Pinguicula agnata *Casper; [GenBank:AF482617]	*Pinguicula agnata *Casper; [GenBank:AF482543.1]
***Pinguicula***	Lentibulariaceae	*Pinguicula alpina *L.; [GenBank:AF531783]	*Pinguicula alpina *L.; [GenBank:AF482618]	*Pinguicula alpina *L.; [GenBank:AF482544.1]
***Pinguicula***	Lentibulariaceae	*Pinguicula lusitanica *L.; [GenBank:DQ010661]	*Pinguicula lusitanica *L.; [GenBank:AF482625.1]	*Pinguicula lusitanica *L.; [GenBank:AF482551.1]
***Plantago***	Plantaginaceae	*Plantago media *L.; [GenBank:AY667474.1]	*Plantago media *L.; [GenBank:AY101920]	*Plantago argentea *Chaix; [GenBank:AJ431056.1]
***Plocosperma***	Plocospermataceae	*Plocosperma buxifolium *Benth.; [GenBank:AJ429315]	*Plocosperma buxifolium *Benth.; T.Borsch, H.Flores, S.Zumaya 377; (BONN); [EMBL:FN794071]	*Plocosperma buxifolium *Benth.; T.Borsch, H.Flores, S.Zumaya 377; (BONN); [EMBL:FN794106]
***Polypremum***	Tetrachondraceae	*Polypremum procumbens *L.; [GenBank:AJ429351.1]	*Polypremum procumbens *L.; [GenBank:AJ430938.1]	*Polypremum procumbens *L.; [GenBank:AJ431063.1]
***Proboscidea***	Martyniaceae	*Proboscidea louisiana *(Mill.) Thell.; [GenBank:AF531809]	*Proboscidea louisiana *(Mill.) Thell.; [GenBank:AJ608573]	*Proboscidea louisiana *(Mill.) Thell.; Kai Müller KM706; BG Bonn 17132; (BONN); [EMBL:FN794107]
***Rehmannia***		*Rehmannia elata *N.E.Br.; Hong-Qing Li 2004-0607; (HSNU); [EMBL:FN773554]	*Rehmannia glutinosa *Steud.; [GenBank:AY423124]	*Rehmannia angulata *(Oliv.) Hemsl.; [GenBank:AJ609145]
***Rhynchoglossum***	Gesneriaceae	*Rhynchoglossum gardneri *Theobald & Grupe; B. Schäferhoff 67; (BONN); [EMBL:FN773555]	*Rhynchoglossum obliquum *Blume; [GenBank:AY423133.1]	*Rhynchoglossum gardneri *Theobald & Grupe; B. Schäferhoff 67; (BONN); [EMBL:FN794108]
***Salvia***	Lamiaceae	*Salvia coccinea *Juss. ex Murr.; [GenBank:AY840147.1]	*Salvia coccinea *Juss. ex Murr.; [GenBank:AY506617.1]	*Salvia guaranitica *A.St.-Hil. ex Benth.; [GenBank:AJ505421.1]
***Schlegelia***	Schlegeliaceae	*Schlegelia parviflora *(Oerst.) Monach.; [GenBank:AJ429345.1]	*Schlegelia parviflora *(Oerst.) Monach.; [GenBank:AJ608570.1]	*Schlegelia parviflora *(Oerst.) Monach.; [GenBank:AJ431057.1]
***Scoparia***	Plantaginaceae	*Scoparia dulcis *L.; E. Fischer 10254; (BONN); [EMBL:FN773556]	*Scoparia dulcis *L.; E. Fischer 10254; (BONN); [EMBL:FN794072]	*Scoparia dulcis *L.; E. Fischer 10254; (BONN); [EMBL:FN794109]
***Scrophularia***	Scrophulariaceae	*Scrophularia chrysantha *Jaub. & Spach; B. Schäferhoff 68; (BONN); [EMBL:FN773557]	*Scrophularia canina *L.; [GenBank:AY423123]	*Scrophularia arguta *[Soland.]; [GenBank:AJ431061]
***Sesamum***	Pedaliaceae	*Sesamum indicum *L.; [GenBank:AJ429340.1]	*Sesamum indicum *L.; [GenBank:AF479010.1]	*Sesamum indicum *L.; [GenBank:AJ609226.1]
***Seymeria***	Orobanchaceae	*Seymeria pectinata *Pursch; [GenBank:AF051999.1]	*Seymeria laciniata *Standl.; [GenBank:EF103898.1]	*Seymeria laciniata *Standl.; [GenBank:EF103820.1]
***Stachytarpheta***	Verbenaceae	*Stachytarpheta cayennensis *(L.C. Rich.) Vahl; E. Martínez S. 37128; (M); [EMBL:FN773558]	*Stachytarpheta cayennensis *(L.C. Rich.) Vahl; [GenBank:AJ608567.1; (M)	*Stachytarpheta cayennensis *(L.C. Rich.) Vahl; [GenBank:AJ299259.1; (M)
***Stemodia***	Plantaginaceae	*Stemodia durantifolia *Sw.; [GenBank:AY492164.1]	*Stemodia glabra *Spreng.; [GenBank:AJ608566.1]	*Stemodia durantifolia *Sw.; [GenBank:AY492225]
***Stemodiopsis***	Linderniaceae	*Stemodiopsis ruandensis *Eb.Fisch.; E. Fischer 10352; (BONN); [EMBL:FN773559]	*Stemodiopsis ruandensis *Eb.Fisch.; E. Fischer 10352; (BONN); [EMBL:794073]	*Stemodiopsis ruandensis *Eb.Fisch.; E. Fischer 10352; (BONN); [EMBL:FN794110]
***Stilbe***	Stilbaceae	*Stilbe ericoides *L.; [GenBank:AJ429350.1]	*Stilbe ericoides *L.; [GenBank:AJ430937.1]	*Stilbe ericoides *L.; [GenBank:AJ431062.1]
***Streptocarpus***	Gesneriaceae	*Streptocarpus bindseili *Eb.Fisch.; [GenBank:AF531810]	*Streptocarpus bindseili *Eb.Fisch,; E. Fischer 1006; Ruanda; (KOBL, BR); [EMBL:794074]	*Streptocarpus bindseili *Eb.Fisch,; E. Fischer 1006; Ruanda; (KOBL, BR); [EMBL:FN794111]
***Tetrachondra***	Tetrachondraceae	*Tetrachondra patagonica *Skotsb.; [GenBank:AJ429352.1]	*Tetrachondra patagonica *Skotsb.; [GenBank:AJ430939.1]	*Tetrachondra patagonica *Skotsb.; [GenBank:AJ431064.1]
***Tetranema***	Plantaginaceae	*Tetranema roseum *(M.Martens & Galeotti) Standl. & Steyerm.; [GenBank:AY667475]	*Tetranema roseum *(M.Martens & Galeotti) Standl. & Steyerm.; [GenBank:AY492192]	*Tetranema roseum *(M.Martens & Galeotti) Standl. & Steyerm.; [GenBank:AY492226.1]
***Thomandersia***	Thomandersiaceae	*Thomandersia hensii *De Wild. Et T. Durand; D. Champluvier 5351; (M); [EMBL:FN773560]	*Thomandersia hensii *De Wild. Et T. Durand; D. Champluvier 5351; (M); [EMBL:794075]	*Thomandersia hensii *De Wild. Et T. Durand; D. Champluvier 5351; (M); [EMBL:FN794112]
***Thunbergia***	Acanthaceae	*Thunbergia alata *Sims; [GenBank:AF531811]	*Thunbergia alata *Sims; [GenBank:AF061820]	*Thunbergia alata *Sims; [GenBank:AJ609131]
***Torenia***	Linderniaceae	*Torenia stolonifera *Boj. Ex Benth.; E. Fischer 10257;(BONN); [EMBL:FN773561]	*Torenia stolonifera *Boj. Ex Benth.; E. Fischer 10257; (BONN); [EMBL:794076]	*Torenia stolonifera *Boj. Ex Benth.; E. Fischer 10257; (BONN); [EMBL:FN794113]
***Utricularia***	Lentibulariaceae	*Utricularia subulata *L.; [GenBank:AF531821]	*Utricularia subulata *L.; [GenBank:AF482676]	*Utricularia subulata *L.; [GenBank:AF482599.1]
***Utricularia***	Lentibulariaceae	*Utricularia multifida *R.Br.; [GenBank:AF531848]	*Utricularia multifida *R.Br.; [GenBank:AF482659]	*Utricularia multifida *R.Br.; [GenBank:AF482583]
***Utricularia***	Lentibulariaceae	*Utricularia biloba *R. Br.; B. Schäferhoff 69; cult. BG Bonn 19853; (BONN); [EMBL:FN773561]	*Utricularia biloba *R. Br.; [GenBank:AF482634]	*Utricularia biloba *R. Br.; [GenBank:AF482561.1]
***Verbena***	Verbenaceae	*Verbena rigida *Spreng.; [GenBank:AF531820]	*Verbena rigida *Spreng.; Kai Müller KM742; BG Bonn 4147; (BONN); [EMBL:794077]	*Verbena rigida *Spreng.; [GenBank:AJ431065]
***Vitex***	Lamiaceae	*Vitex trifolia *L.; [GenBank:AB284175.1]	*Vitex trifolia *L.; [GenBank:AJ505539.1]	*Vitex trifolia *L.; [GenBank:AJ505416.1]
outgroup				
***Coffea***	Rubiaceae	*Coffea arabica*; [GenBank:EF044213]	[GenBank:EF044213]	[GenBank:EF044213]
***Nicotiana***	Solanaceae	*Nicotiana tabacum*; [GenBank:NC001879.2]	[GenBank:NC001879.2]	[GenBank:NC001879.2]
***Solanum***	Solanaceae	*Solanum tuberosum*; [GenBank:DQ231562]	[GenBank:DQ231562]	[GenBank:DQ231562]

Key: Voucher information (collector and number, garden accession number if from living collection, herbarium acronym in braces) is provided for sequences newly generated in this study.

### Amplification and sequencing

Total genomic DNA was isolated using the AVE Gene Plant Genomics DNA Mini Kit (AVE Gene, Korea), according to the manufacturer's protocol. As phylogenetic markers, the *trnK *intron including the coding *matK*, the *trnL-F *region, and the *rps*16 intron were amplified using standard PCR protocols. Primers used for amplification and sequencing are given in Table 2. Reactions were performed in 50 μl volumes containing 2 μl template DNA (10 ng/μl), 10 μl dNTP mix (1.25 mM each), 2 μl of each forward and reverse primer (20 pm/μl), and 0.25 μl Taq polymerase (5 U/μl, Peqlab). Thermal cycling was performed on an Biometra T3 thermocycler using the following PCR profiles: 1:30 min at 96°C, 1 min at 50°C, 1:30 min at 72°C, 35 cycles of 30 sec at 96°C, 1 min at 50°C, 1:30 min at 72°C, and a final extension time of 10 min at 72°C for the *trnK *intron; 35 cycles of 1 min at 94°C, 1 min at 52°C and 2 min at 72°C, followed by a final extension time of 15 min at 72°C for the *trnL-F *region; 1:30 min at 94°C, 30 cycles of 30 sec at 94°C, 30 sec at 56°C and 1 min at 72°C, and a final extension time of 15 min at 72°C for the *rps16 *intron. Fragments were gel-purified on a 1.2% agarose gel (Neeo-agarose, Roth), extracted with the Gel/PCR DNA Fragments Extraction Kit (AVE Gene, Korea) and sequenced on an ABI3730XL automated sequencer using the Macrogen sequencing service (Macrogen Inc., Seoul, Korea). Pherogram editing and contig assembly was done manually.

**Table 2:** Primers used in the present study

Name	Sequence 5'-3'	Design
trnK3914Fdi	GGGGTTGCTAACTCAACGG	Johnson and Soltis [120]
LE1R	ATAGAAATAGATTCGTTC	Müller et al. [13]
LE4R	TTCGCCTGAAAATCCGTAACC	Müller et al. [13]
LE5R	CAAGGTTCCTTGCRCCAACC	this study
ACmatK500F	TTCTTCTTTGCATTTATTACG	Müller and Borsch [121]
LindmatK1714R	CTCCAAAGAAAGYCAGTTCCTCTT	this study
LindmatK1580F	TCAATTCATTCAACWTTTCCC	this study
LE2F	TGGTACGGAGTCAAAKTC	Müller et al. [13]
trnK2R	AACTAGTCGGATGGAGTAG	Johnson and Soltis [120]
trntC2	TATGGCGAAATTGGTAGACGC	this study
trntF	ATTTGAACTGGTGACACGAG	Taberlet et al. [122]
rpsF	GTGTGTAGAAAGCAACGTGCGACTT	Oxelman et al. [123]
rpsR2	TCGGGATCGAACATCAATTGCAAC	Oxelman et al. [123]

### Addition and analysis of GenBank sequence data

We additionally took *rbcL *and *ndhF *sequences (see Additional file 1, Table S1) for relevant taxa from GenBank, and in a separate set of analyses combined them with our three marker dataset. Taxon sampling of these four- and five-region datasets was adapted to include only taxa with all regions present.

Because the position of *Hydrostachys *remained inconsistent in previous studies, all sequences from that genus existing in GenBank were blasted against the entire data of GenBank via blastn [61]. Additionally, *trnK*/*matK*, *rps16 *and *trnL-F *sequences for *Hydrostachys *from a collection independent from those previously used [31,33,62,63] were generated; all sequences used, including voucher information, are given in Table 1. The newly generated *Hydrostachys matK *sequence was aligned to an existing angiosperm *matK *alignment [35] and subjected to parsimony analysis.

### Alignment and indel coding

DNA sequences were manually aligned in PhyDE [64], taking microstructural changes into account as outlined elsewhere [58,65]. Regions of uncertain homology were excluded from phylogenetic analyses. For maximum parsimony (MP) analyses and Bayesian Inference of Phylogeny (BI), indels were coded according to simple indel coding (SIC) [66] using the program SeqState [67].

### Parsimony analyses

Searches for the shortest tree were performed using the parsimony ratchet approach implemented in PRAP2 [68] using the following settings: 10 random addition cycles with 200 ratchet replicates, setting the weight for 25% of the characters to 2. The files generated were executed in PAUP* v4.0b10 [69]. Bootstrapping was performed with 10,000 replicates, each using TBR branch swapping and holding only one tree [70]. We measured the additional information provided by SIC-coded indels by the difference in decay indices (computed with PRAP2) for each node, comparing analyses with and without indels.

### Bayesian Inference of Phylogeny

Bayesian inference (BI) of phylogeny was done with help of MrBayes v3.1.2 [71]. The model of best fit for the combined dataset as well as for each of the three partitions (*trnK*/*matK*, *rps16 *and *trnL-F) *was found to be GTR+G+I model was found as the optimal one using jModelTest v.0.1.1 [72]. The indel partition was co-analyzed together with the DNA partition, with the restriction site (binary) model applied to the gap characters and the ascertainment (coding) bias set to "variable". Default priors were used, i.e. flat dirichlets (1.0, 1.0) for state frequencies and instantaneous substitution rates, a uniform prior (0.0, 50.0) for the shape parameter of the gamma distribution, a uniform prior (0.0, 1.0) for the proportion of invariable sites, a uniform topological prior, an exponential prior Exp (10.0) for branch lengths. Four categories were used to approximate the gamma distribution. Two runs with 5 million generations each were run, and four chains were run in parallel for each run, with the temperature set to 0.2. The chains were sampled every 100th generation, and the burnin was set to 5000. To check for convergence of the independent runs under a given model, it was ensured that the plots of both runs indicated that the stationary phase was reached, that the potential scale reduction factor approached 1 for all parameters, and that no supported conflicting nodes were found among the consensus trees generated from each run. Convergence and effective sampling sizes (ESS) of all parameters were assessed with halp of Tracer v1.5 [73].

### Maximum likelihood analyses

For maximum likelihood (ML) analyses RAxML v7.0.0 [74] was used. During the search for the best tree, the GTRGAMMA model was used, while the slightly simpler GTRCAT model was employed by RAxML during the 500 bootstrap replicates. Support values from all types of analysis were mapped on the tree topology from the ML analysis and conflicting nodes were identified with help of TreeGraph2 [75].

### Topological tests

Topological tests were used to see whether alternative topologies could be rejected with confidence. Specifically it was tested whether evidence against Byblidaceae being sister to Lentibulariaceae was strong. Under parsimony, the Templeton and Winning-sites (sign) tests were used ("NonparamTest" option in Paup*), while under the likelihood criterion, the Approximately Unbiased test (AU-Test) [76] along with the more classical Shimodaira-Hasegawa test (SH-test [77]), as implemented in consel 0.1j [78], were employed.

### Ancestral state reconstruction

We inferred ancestral states for ten selected morphological characters. Information on character states was compiled from different sources [79,1,27,80] and is given in Table 3. We took the fully resolved best tree from the RAxML search, and traced the evolution of these characters on that topology via maximum likelihood, using the "multistate" command in BayesTraits [81].

**Table 3:** Morphological characters traced in the present study

Taxon/character	1	2	3	4	5	6	7	8	9	10
**Outgroup**	0	0	0	?	0	0	?	?	0	0
**Plocospermataceae**	0/1	0	0	0	0	0	0	0	0	0
**Carlemanniaceae**	1	0	2	0	0	0	0	0	0	0
**Oleaceae**	1	0	2	0	0	0	0	0	0	0
**Tetrachondraceae**	1	0	1	0	0	?	0	?	0	0
**Calceolariaceae**	1	1	2	0	1	1	0	1	0	0
**Gesneriaceae**	0	1	1	0	1	1	0	1	0	0
**Plantaginaceae**	0	1	0/1/2	0	0	1	1	?	0	0
**Gratiolaceae**	0	1	1	0	0	1	1	0	0	0
**Scrophulariaceae**	0	1	1	0	0	1	1	0/1	0	0
**Byblidaceae**	0	0	0	0	0	1	1	0	1	0
**Linderniaceae**	0	1	1	1	0	1	1	0/1	0	0
**Stilbaceae**	0	1	1	0	0	1	1	0	0	0
**Lamiaceae**	0	1	1	0	0	1	1	0	0	0
**Mazoideae**	0	1	1	0	0	1	1	0	0	0
**Phrymoideae**	0	1	1	0	0	1	1	0	0	0
**Paulowniaceae**	0	1	1	0	0	1	1	0	0	0
**Rehmannia**	0	1	1	0	0	?	1	1	0	0
**Orobanchaceae**	0	1	1	0	0	1	1	1	0	1
**Thomandersiaceae**	0	1	1	0	0	1	1	0	0	0
**Pedaliaceae**	0	1	1	0	0	1	1	0	0	0
**Bignoniaceae**	0	1	1	0	0	1	1	0	0	0
**Verbenaceae**	0	1	1	0	0	1	1	0	0	0
**Schlegeliaceae**	0	1	1	0	0	1	1	0	0	0
**Martyniaceae**	0	1	1	0	0	1	1	0	0	0
**Acanthaceae**	0	1	1	0	0	1	1	0	0	0
**Lentibulariaceae**	0	1	2	0	0	1	1	0	1	0

Key: **1: **merosity 0 = pentamerous 1 = tetramerous; **2: **symmetry 0 = polysymmetric 1 = monosymmetric; **3: **number of stamens 0 = 5 1 = 4 2 = 2; **4: **geniculate stamens 0 = absent 1 = present; **5: **pair flowered cymes 0 = absent 1 = present; **6: **Anthraquinones from shicimic acid metabolism 0 = absent 1 = present; **7: **biosynthetic route II decarboxylated iridoids 0 = absent 1 = present; **8: **alveolated seeds 0 = absent 1 = present; **9: **Carnivory 0 = absent 1 = present; **10: **Parasitism 0 = absent 1 = present.

## Results

### Sequence statistics and results from tree searches

**Figure 2:**
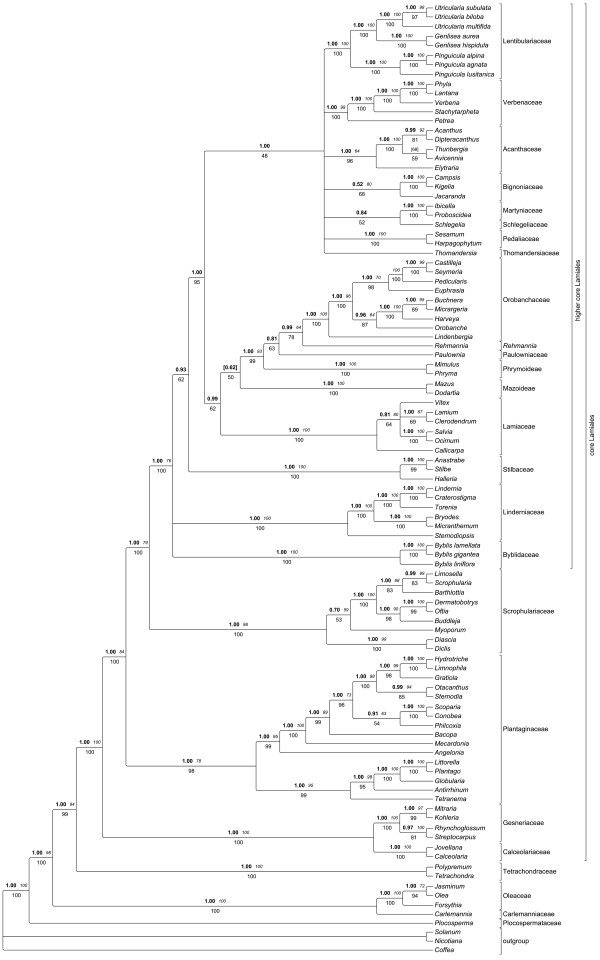
**Phylogeny of Lamiales inferred from parsimony, likelihood and Bayesian analysis of sequences from plastid *trnK*/*matK*, *trnL-F *and *rps*16**. Topology from the maximum likelihood tree depicted, collapsing nodes not supported by > = 50% in at least one of the three analyses. Bold numbers above branches are posterior probabilities from Bayesian inferences, italic numbers above branches are MP bootstrap values, number below branches indicate ML bootstrap proportions. Numbers in brackets indicate that the respective node was not supported by all three methodological approaches. The bracketed number then indicates the strongest support found for any node that contradicts the shown node [69]. Familial annotation according to APG III [28]. For Phrymaceae monophyly is not confirmed, so subfamilies are annotated; *Rehmannia *is currently not assigned to a family.

**Figure 3:**
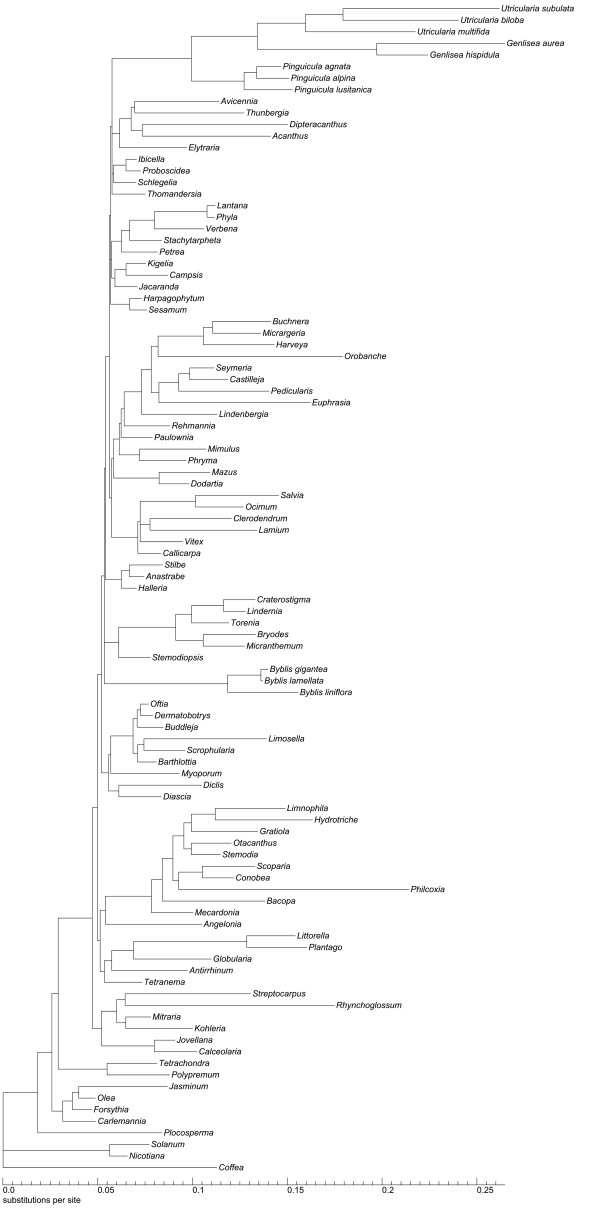
**Phylogram from Bayesian Inference of phylogeny with branch lengths giving the relative substitution rates using the GTR+G+I model**.

Sequences of *trnK*/*matK*, *trnL-F *and *rps16 *yielded an alignment of 7809 characters, of which 1739 were excluded from subsequent analysis because of uncertain homology. The alignment is available from TreeBase (http://purl.org/phylo/treebase/phylows/study/TB2:S10963); detailed sequence statistics are given in Table 4. Consensus trees from parsimony analyses were well resolved and supported. The MP trees from substitutions only were 13118 steps long (CI 0.419, RI 0.504,), those based on substitution and indel characters had a length of 14719 steps (CI 0.453, RI 0.507,). Comparison of decay values of substitution data versus substitutions plus SIC-coded indels showed higher decay values for most nodes when indel information was included (see Additional file 2, Figure S1). Trees from coding *rbcL *and *ndhF *seqences were far less resolved than those from our three marker combined analysis (Additional file 3 Figure S2 and Additional file 4, Figure S3). The tree topology from the ML analysis is shown in Figure 2, collapsing nodes support by less than 50% in at least one of the tree methodological approaches. BI and ML trees generally showed slightly higher resolution and statistical support than trees from MP searches. Effective sampling sizes (ESS) of all parameters from the Bayesian analysis were > 150. A phylogram from BI with branch lengths indicating relative substitution rates is given in Figure 3.

**Table 4:** Sequence statistics for the rapidly evolving chloroplast markers used

charset	#chars	#chars*	length range	mean	S.D.	%divergence*	S.E.*	%variable*	%informative*	%GC
**dataset**	7809	6070	2211-4503	3.926.44	482.561	10.15	0.187	51.417	36.063	34.212
**trnK/matK**	3699	3035	454-2645	2.228.78	446.491	10.367	0.264	60.362	43.229	43.229
**trnLF**	1997	1577	489-1104	882.881	72.353	9.086	0.402	40.076	28.155	28.155
**rps16**	2113	1458	0-929	814.772	122.607	10.792	0.464	45.062	29.698	29.698

* calculated based on the alignment with hotspots excluded

**Standard errors calculated based on 100 bootstrap replicates**.

Key: Characters = number of characters in the alignment matrix; Length range = actual sequence length in nucleotides (including hotspots; minimal and maximal value observed); SD = standard deviation of mean length; S.E. = Standard error; % divergence (range) = pairwise sequence distance in percent (uncorrected p distance, overall mean); % variable = percentage of variable positions; % informative = percentage of parsimony informative positions; % GC = GC content.

### Resolution of the backbone of the Lamiales phylogeny

The precise branching pattern of the nine first-branching families in the Lamiales tree (Plocospermataceae, Carlemanniaceae, Oleaceae, Tetrachondraceae, Calceolariaceae, Gesneriaceae, Plantaginaceae (incl. Gratioleae), Scrophulariaceae) is inferred with very high or maximum (most cases) support (Figure 2). A total of 16 nodes determining this branching pattern among families along the spine of the basal Lamiales grade receive very high or maximum support by all (most cases) or at least two out of three inference methods. An additional 19 of the nodes indicating delimitation and relative position of the remaining 15 more derived families receive very high or maximum support by at least one out of three analytic approaches.

### Phylogenetic position of *Hydrostachys*

In our blastn searches, all sequences (*rbcL*, *atpB*, 18s rDNA, 26s rDNA, *ndhF*, *matK*) reached highest similarity scores to other *Hydrostachys *sequences, followed by sequences from Cornales taxa (Hydrangeaceae, Cornaceae, Loasaceae), with the exception of the *matK *sequence of *Hydrostachys multifida *(AY254547) of Hufford et al. [82] used in the study of Burleigh et al. [31]. This sequence showed highest similarity with *Hydrangea hirta *and a number of sequences from *Avicennia*. When included in the present *trnK/matK *alignment, the high similarity of sequence AY254547 to *Avicennia *is obvious. A blast search of the newly generated *matK *sequence of *Hydrostachys *[EMBL: FN8112689] resulted in best matches with taxa from Cornales. Aligning and analyzing the newly generated *trnK*/*matK*, *rps16 *and *trnL-F *sequences, *Hydrostachys *is resolved outside Lamiales. Parsimony analysis of the newly generated *matK *sequence in the context of the angiosperm *matK *data set [35] evidently places the newly generated *matK *sequence of *Hydrostachys *outside Lamiales, although its precise position within asterids remains unresolved in the 50%-majority-rule-bootstrap tree (Additional file 5, Figure S4).

### Position of carnivorous lineages

In neither the Bayesian nor the maximum likelihood analysis Byblidaceae were found closely related to Lentibulariaceae. In MP analyses, the position of Byblidaceae receives no bootstrap support; interestingly, however, the strict consensus from all shortest trees depicts Byblidaceae as sister to Lentibulariaceae, regardless of the inclusion of indels. Because of this incongruence, albeit unsupported, topological tests were employed to further investigate the position of Byblidaceae. Under a parsimony framework, the Templeton and sign tests find the ML topology (Byblidaceae not closely related to Lentibulariaceae) not to be significantly less parsimonious than the shortest tree (Table 5), indicating that even under parsimony there is no significant evidence against the ML position of Byblidaceae or for its sister-group relationship to Lentibulariaceae. The AU-Test and SH-Test indicate that a sister-group relationship of Byblidaceae and Lentibulariaceae is significantly less likely than the maximum likelihood and Bayesian consensus topology.

**Table 5:** Results from topology tests

		Templeton	Winning-sites	Approxiomately Unbiased	Shimodaira-Hasegawa
**topology**	Length	P	P	P	P
**tree 1**	13123	0.2971	0.4049	1.000	0.994
**tree 2**	13118			5e-004	0.006

Key: Maximum Parsimony: Templeton- and Winning-sites tests. Tree 1: optimal tree from RAxML search (Figure 2), tree 2: optimal tree from MP ratchet search, where Byblidaceae appear as sister to Lentibulariaceae. P = Approximate probability of getting a more extreme test statistic under the null hypothesis of no difference between the two trees (two-tailed test). The shortest tree (tree 2) is not significantly different from the ML topology (tree 1, Figure 2). Maximum Likelihood: Approximatly Unbiased- and Shimodaira-Hasegawa tests. The ML topology (tree 1, Figure 2) is significantly different from and more likely than the MP alternative (tree 2).

### Results from ancestral state reconstruction

**Figure 4:**
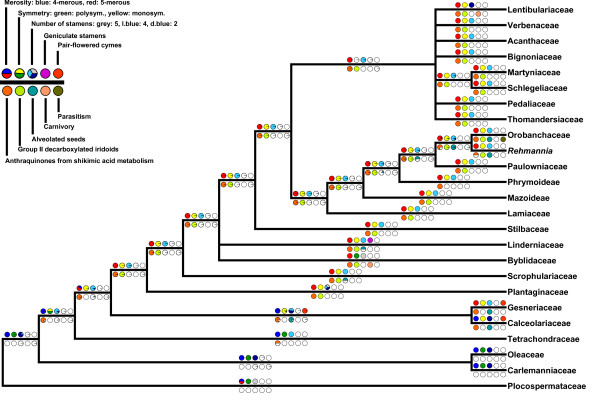
**Evolution of selected morphological characters in Lamiales**. ML ancestral state reconstruction on the ML topology (Figure 2) simplified to represent families by only one OTU and collapsing nodes not supported by > = 50% in at least one of the analyses. Pie charts give probabilities of character states; white indicates absence in case of binary (presence-absence) characters, while color indicates presence. Otherwise, colors indicate states as shown in legend.

Ancestral state reconstruction indicated the probabilities of the individual character states to be expected along branches as shown in Figure 4.

## Discussion

Lamiales sensu APGIII [28] (including Carlemanniaceae and Plocospermataceae) receive maximal support in the present study which is the first to sample taxa from these two families in a multigene study; a single gene study [36] did not provide support for the branching order of the early branching lamialean families.

### The phylogenetic position of *Hydrostachys*

*Hydrostachys *as a rheophyte with tuber-like rhizomes, fibrous roots, and no stomata is a morphologically highly aberrant genus [32], which has always hampered inference of its phylogenetic affinities based on morphology. Embryological characters such as endosperm development and the apical septum in the ovary [83] might be interpreted as supporting a placement of *Hydrostachys *in Lamiales [31]. The first molecular study, however, placed it within Cornales [34]. In all previous phylogenetic studies, the genus was found on a long branch, indicating strongly elevated substitutional rates - a fact that could have misled previous phylogenetic inferences [33]. Burleigh et al. [31] recently used a 5-gene data matrix to infer an angiosperm phylogeny, and resolved *Hydrostachys *as nested in Lamiales, branching right after Oleaceae. Results from our re-sequencing and re-analysis, along with a blast screening of existing GenBank sequences, strongly suggest that this placement most likely was due to an erroneous *matK *sequence used in their study. That sequence was first published by Hufford et al. [82] but is identical to one published earlier by Hufford et al. [62], although citing a different voucher. Interestingly, Burleigh et al. [31] report that the 3-gene matrix (*rbcL*, *atpB*, 18S) places *Hydrostachys *in Cornales, while in the 5-gene matrix (additional *matK *and 26S data), *Hydrostachys *is found in Lamiales. The authors suggest the *matK *sequence to be the driving force for this result. Indeed, the most likely incorrect *matK *sequence misinforms phylogenetic inference, even though only one out of five genes provides the erroneous signal. If nothing else, this demonstrates the strong phylogenetic signal and potential of *matK *for phylogenetic analyses at the given phylogenetic depth. Phylogenetic reconstruction using our newly generated sequences in the context of the three-marker matrix compiled here and in the context of the angiosperm *matK *alignment clearly places *Hydrostachys *outside Lamiales, which is consistent with earlier findings [36,84,85] and with the analysis of two unpublished *matK *sequences by Kita and Kato (AB038179, AB038180).

### A robust hypothesis on the basal grade in Lamiales

The Central American Plocospermataceae branch first in Lamiales (Figure 2), a scenario also found earlier in all studies that sampled this monotypic family [26,35,36]. A clade consisting of Carlemanniaceae plus Oleaceae branches second. A close relationship between these two families was found weakly supported (64% BS) previously [36] based on *rbcL *sequences, and was also observed in a study dealing with plastome rearrangements in Oleaceae [35], when Carlemanniaceae appeared sister to Oleaceae despite being set to as outgroup. We find the sister group relationship between Carlemanniaceae and Oleaceae with maximum support.

Tetrachondraceae are recovered with maximum support in all three analyses as third branch in Lamiales. While this relationship has been observed previously [36,26], statistical support for it has increased significantly in our study (59% MP BS support in Savolainen et al. [36] versus PP 1.00, 100% ML BS, 94% MP BS, support in our tree). The family comprises two genera, *Tetrachondra *and *Polypremum*, both of which were sampled here. The genus *Tetrachondra *has a disjunct distribution (New Zealand/South America) and comprises the two aquatic or semi-aquatic species, while the monotypic *Polypremum *is found from southern U.S. to the northern part of South America.

### Relationships within core Lamiales

The core Lamiales (sensu [35], all Lamiales excluding Carlemanniaceae, Oleaceae, Plocospermataceae, and Tetrachondraceae; Figure 2) are unambiguously recovered by our analysis. As first branch within this core group a maximally supported clade composed of Calceolariaceae and Gesneriaceae (Figure 1f, g) is found. The phylogenetic affinities of both families had remained unclear so far [45,38,2] but both share the presence of cornoside and absence of iridoids [86]. Gesneriaceae are a large (ca. 3200 species), predominantly pantropical family of herbaceous perennials (rarely woody shrubs and small trees), about one fifth of them growing as epiphytes [87]. In contrast to many other lamialean families, molecular phylogenetics confirmed their traditional circumscription, as proposed by Bentham in 1876 [88].

#### Plantaginaceae

Next in the basal grade of core Lamiales is a clade comprising Plantaginaceae as currently defined [28] (PP 1.00, 100% ML BS, 84% MP BS), in which a major split separates two groups from each other. All former studies focusing on Plantaginaceae relationships found a major dichotomy within this family [38,22,39,89]. Rahmanzadeh et al. [2] argued that the finding of a well supported clade including genera from Gratioleae together with unclear relationships of this group to other families is handled best with the recognition of a separate family. Thus, Gratiolaceae were resurrected [2]. Current phylogenies allow both the recognition of two families, as well as the treatment of Plantaginaceae with two major subfamilies. Since the taxon sampling is still far from being complete, and clear morphological characters for either of the groups are lacking, we solely accept Plantaginaceae throughout this manuscript. Rahmanzadeh et al. [2] tentatively assigned 36 genera to their Gratiolaceae, 13 of which were included in our phylogenetic study. Among the genera proposed to be part of Gratiolaceae, the widespread genus *Limosella *was found in Scrophulariaceae [22,39], and the present analysis confirms placement of *Limosella *in Scrophulariaceae. *Stemodiopsis *is found in Linderniaceae, while *Lindenbergia *is sister to the remaining Orobanchaceae. According to Olmstead et al. [38] and Rahmanzadeh et al. [2], Angelonieae (two genera: *Angelonia *and *Monopera*) appears closely related to Gratioleae. Gratioleae have an integument 3-6 cells across, with large, transversely elongated endothelial cells in vertical rows; this causes its seeds to have longitudinal ridges. The exotestal cells have hook-like thickenings [1]. Stevens et al. [1] suggest Angelonieae (integument 5-12 cells across) should also be included in Gratioleae. However, a denser taxon sampling will be needed to further test what belongs in this clade-regardless of the taxonomic level on which it might be recognized.

#### Scrophulariaceae

Scrophulariaceae in their new circumscription, including former Buddlejaceae and Myoporaceae, are the sister to all other higher core Lamiales (PP 1.00, 100% ML BS, 79% MP BS). This was already indicated by previous studies [2,39] and is confirmed here with high confidence. A vastly expanded circumscription of Scrophulariaceae that was presented as a possibility in APGIII [28] would thus mean that all higher core Lamiales would have to be included in order to respect the principle of monophyletic families. Such a classification would have to include a morphologically very heterogeneous assemblage of lineages with more than 17.000 species and does therefore not appear as very helpful.

### Higher core Lamiales (HCL) and the evolution of carnivory

The remaining families Acanthaceae, Bignoniaceae, Byblidaceae, Lamiaceae, Lentibulariaceae, Linderniaceae, Orobanchaceae, Paulowniaceae, Pedaliaceae, Phrymaceae, Schlegeliaceae, Stilbaceae, Thomandersiaceae, and Verbenaceae form a clade strongly supported by BI (PP 1.00) and ML (100% ML BS) analysis, but only moderately supported (76% MP BS) in MP trees (referred to as "higher core Lamiales", or HCL clade, in the following). There is no morphological synapomorphy known for this clade.

A monophyletic origin of carnivory in Lamiales has been discussed since the introduction of molecular phylogenetics to the field of angiosperm systematics (see chapter on Lamiales in [90]). In the earliest analyses of *rbcL *sequences, the genus *Byblis *was found sister to Lentibulariaceae, but this placement gained only weak statistical support [19]. Later, an analysis of three coding plus three non-coding chloroplast markers [26] found Byblidaceae as sister to Lentibulariaceae with 65% jackknife support. This is the highest statistical support ever reported for this relationship, but only one *Byblis *species and one *Pinguicula *species were sampled in that study.

Based on our data, a close relationship of carnivorous Byblidaceae and Lentibulariaceae is extremely unlikely. The placement of Byblidaceae next to Lentibulariaceae, as found in previous studies and even in single MP tree topologies of the current study, has been rejected at highest significance levels by our topological tests and is contradicted with substantial statistical support by our ML and BI trees. It might be due to long branch attraction, to which MP is much more susceptible than the other two approaches [91].

Accordingly, carnivory evolved at least twice within Lamiales, in congruence with Müller et al. [13]. Our data still do not provide enough resolution to identify the immediate sister group of Lentibulariaceae. The family appears in a weakly supported group together with Acanthaceae, Thomandersiaceae and Martyniaceae/Schlegeliaceae and Bignoniaceae, Pedaliaceae and Verbenaceae. An earlier study, sampling only one species from Lentibulariaceae (*Pinguicula*), found *Elytraria *(Acanthaceae) as sister to Lentibulariaceae [39] with 52% parsimony BS. In contrast, the monophyly of Acanthaceae, including *Elytraria*, was strongly supported in a more recent study sampling 85 taxa from Acanthaceae [92]. In congruence with that, we find *Elytraria *sister to remaining Acanthaceae.

The lack of resolution in higher core Lamiales still hampers a clear identification of the precise degree of relatedness to Martyniaceae, two strongly glandular members of which (*Ibicella *and *Proboscidea*) have been reported to attract and catch numerous arthropods, and thus have been classified as "protocarnivorous". Recent tests for protease activity of glands of the two respective genera were negative [93]; however, putatively mutualistic arthropods have been reported to be associated with each genus [94], from which the plant might benefit in a manner similar to the symbiosis observed in the African *Roridula *(Roridulaceae, Ericales) [93].

Next relatives to the supposedly carnivorous or "protocarnivorous" genus *Philcoxia *are found in Gratioleae, as previously suggested [21]. Without any doubt, Gratioleae have no close connection to Lentibulariaceae, despite some morphological similarity. Should further tests identify *Philcoxia *as a truly carnivorous plant, this would be the third independent origin of the syndrome within the order.

### Further insights into the family circumscriptions in higher core Lamiales

#### Linderniaceae

The exact position of Linderniaceae within higher core Lamiales remains unclear. It is found unresolved in tritomy together with Byblidaceae and a clade including Acanthaceae, Bignoniaceae, Lamiaceae, Lentibulariaceae, Martyniaceae, Orobanchaceae, Paulowniaceae, Pedaliaceae, Phrymaceae, Schlegeliaceae, Stilbaceae, Thomandersiaceae, and Verbenaceae. Only the maximum likelihood tree depicts Linderniaceae and Byblidaceae forming a poorly supported clade. The centers of diversity of this family are in Southeast Asia and tropical Africa. Among them, desiccation tolerant plants like *Craterostigma *are found.

#### Stilbaceae and remaining families

Within the remaining families, the African Stilbaceae branch first; this scenario gains convincing support from Bayesian Inference (PP 0.93), weak support from ML bootstrapping (62% ML BS), and lacks parsimony bootstrap support. Molecular phylogenetic studies had expanded the traditional circumscription of Stilbaceae [38,39,95,96] to 11 genera (3 of which we sampled here) with a predominantly South African distribution. Only *Nuxia *extends to tropical Africa and the Arabian Peninsula.

One of two major clades in this assembly comprises Lamiaceae, Phrymaceae, Paulowniaceae, *Rehmannia*, and Orobanchaceae. Although this clade also was recovered previously [39], this is the first time it receives support from BI and ML. Within that group, Lamiaceae are sister to the remaining taxa, supported by 50% ML BS (our study), and PP 0.92 and 58% MP BS value [39]. We find subfamily Mazoideae of Phrymaceae sister to a clade including *Paulownia*, Phrymaceae subfamily Phrymoideae, *Rehmannia *and Orobanchaceae. Herein, *Rehmannia *is weakly linked to Orobanchaceae, while the relationship between *Paulownia *and Phrymoideae remains unresolved. Previous studies dealing with the next relatives of Orobanchaceae found either *Paulownia *[38], or *Phryma *and *Paulownia *together, but as unresolved tritomy [26], or *Mimulus *and *Paulownia *as successive sisters to Orobanchaceae [2] but did not include *Rehmannia *and/or *Triaenophora*.

With regard to Orobanchaceae relationships, the most extensive sampling in terms of both taxa and character number are that of Xia et al. [43] and Albach et al. [50]. The authors found *Rehmannia *and *Triaenophora *together as sister clade to Orobanchaceae, which should either be included in Orobanchaceae, as suggested by Albach et al. [50], or be recognized as a new family. As a morphological synapomorphy, Orobanchaceae, *Rehmannia *and *Triaenophora *share alveolated seeds [43]. Although a well resolved phylogeny of Orobanchaceae exists, it still remains to be tested using plastid sequence data whether the non-parasitic *Lindenbergia *alone is sister to the remaining Orobanchaceae, or if *Lindenbergia *plus the hemiparasitic genera *Siphonostegia*, *Schwalbea*, *Monochasma*, *Cymbaria *and *Bungea *are in the respective position [49].

Including taxa from both subfamilies of Phrymaceae in a context of putative relatives, no evidence for the monophyly of Phrymaceae was found [37,39]. Only Beardsley and Olmstead [51] found weak support for a monophyletic Phrymaceae, but this result is probably due to the specific sampling used. In that study [51], chloroplast data alone did not support this clade, while nuclear data and the combined analysis did so. The incongruence might be caused by a plastid-nuclear genome incongruity, which must be confirmed by additional data.

The two subfamilies of Phrymaceae, Phrymoideae and Mazoideae, do not form a clade in any of the trees in Xia et al. [43] or Albach et al. [50], and the branching order of Mazoideae, Phrymoideae and *Paulownia *is inconsistent in different analyses of these studies. Hence, the authors abstain from assigning these groups to families. In the light of our data we suggest to segregate Mazoideae from Phrymaceae and elevate it to family rank.

The position of Lamiaceae distinct from Verbenaceae (Figure 2) is an important and noteworthy finding. It ends a century-old discussion on close relationships of a Lamiaceae-Verbenaceae complex [88,97,98]. Molecular phylogenetic analysis rather concluded that Lamiaceae may not be monophyletic with respect to Verbenaceae [99]. However, analyses of *rbcL *[100,99] were not conclusive about their relationships and even a combined *matK/trnK *analysis [2] did not provide sufficient support for Lamiaceae and Verbenaceae.

The families Acanthaceae, Bignoniaceae, Lentibulariaceae, Martyniaceae, Pedaliaceae, Schlegeliaceae, Thomandersiaceae, and Verbenaceae form a clade in our Bayesian and ML analyses (PP 1.00, ML BS 48%). For all families for which more than one taxon was sampled, monophyly is confirmed, but there is only little resolution of intra-familial relationships in that clade, especially in MP trees. In the work of Oxelman et al. [39], a corresponding clade was found, including the families mentioned above, except Pedaliaceae. We find weak support for Schlegeliaceae to be sister to Martyniaceae, while Oxelman et al. [39] found Martyniaceae, Verbenaceae and Schlegeliaceae in a clade (PP 0.82). Wortley et al. [42] found *Thomandersia *weakly linked to Schlegeliaceae, however, our data do not exhibit evidence for support such a relationship. A close examination of the floral anatomy of *Thomandersia *[101] could not improve the knowledge on its relationships.

### Implications for the evolution of floral symmetry and other characters

Within Lamiales, both polysymmetric and monosymmetric (zygomorphic) flowers occur. Next to the typical pentamerous flowers, some groups exhibit tetramerous morphology. With the most highly resolved phylogeny of Lamiales to date, the evolution of floral symmetry and flower merosity within the order can be studied in more detail than previously possible. Assuming the ancestral asterid flower to be pentamerous and polysymmetric, Plocospermataceae as the most basal family of Lamiales, share this plesiomorphic character state (Figure 4). Regarding the evolution of tetramery, there are two possible scenarios. In the first, tetramery evolved once after the branching of Plocospermataceae in Lamiales, with two reversals to pentamery in both Gesneriaceae and then independently in all Lamiales branching after the Calceolariaceae/Gesneriaceae clade, this possibility is the one which is favoured by our ML ancestral state reconstruction. In the second scenario, tetramery evolved three times independently in (i) Oleaceae/Carlemanniaceae clade, (ii) Tetrachondraceae, and (iii) Calceolariaceae. Both options require three changes in flower merosity, and thus are equally parsimonious. However, there are details in floral development that differ among the tetramerous families. In Oleaceae, sepals are initiated in orthogonal positions, and petals are in diagonal position, whereas in Tetrachondraceae, sepals are initiated in diagonal, and petals in orthogonal position [102]. Initiation in Calceolariaceae follows that in Oleaceae; data for Carlemanniaceae are missing. Because tetramery in the early branching lineages of Lamiales is different for each group on more detailed level, independent gains seem more likely than a general shift towards tetramery and two independent reversals to pentamery. Tetramerous flowers are also found in the more derived Gratioleae, Veroniceae and Plantagineae (Plantaginaceae). Based on mixed evidence for fusion and loss of flower parts in these groups, multiple origins of tetramery within Plantaginaceae have been assumed. For the Plantaginaceae, Bello et al. [103] hypothesize two shifts from pentamery to tetramery: (i) in *Amphianthus*, which has recently been shown to be nested in *Gratiola *[89], and (ii) in a clade consisting of *Aragoa*, *Plantago *and *Veronica*. An independent shift to tetramery has been suggested by Albach et al. [104] based on loss of a sepal in Veroniceae and fusion in *Plantago *and *Aragoa*. But in these taxa the upper lip is composed out of two petals. Evidence for this is vascularization with two midribs, teratologic, pentamerous flowers, and an evolutionary row from pentamerous to tetramerous flowers within this tribe [98,82]. The evolution of flower symmetry can be easily reconstructed. Lamiales descended from a polysymmetric ancestor, and early branching lineages in Lamiales share this character state. After branching of Tetrachondraceae, the ancestor of the following taxa once acquired monosymmetric flowers, accompanied by a reduction from five stamens to four stamens plus one staminode. There are multiple transitions back to actinomorphic flowers in Lamiales, e.g. in the case of *Plantago *(Plantaginaceae) [103,105], in some taxa in Lamiaceae, Scrophulariaceae, Gesneriaceae, and in all Byblidaceae. The corolla of Byblidaceae is treated here as actinomorphic, although the curved stamens introduce a slight element of zygomorphy.

#### Further morphological characters

Several morphological or biochemical characters lend further support to some of our hypothesized phylogenetic relationships in Lamiales. Carlemanniaceae and Oleaceae share the characteristic of having only two stamens, while the first-branching Plocospermataceae have five stamens, and the lineages branching later in the evolution of Lamiales generally have four stamens. The sister-group relationship between Calceolariaceae and Gesneriaceae is further confirmed by two morphological characters shared by these families (see Figure 4): (i) the thyrsic inflorescence with pair flowered cymes, and (ii) aulacospermous alveolated seeds [102]. Aulacospermous seeds are otherwise only found in Linderniaceae (*Crepidorhopalon, Hartliella*). However, an aberrant type of aulacospermous seeds is found in some genera of Scrophulariaceae s.str.. Here not all cells of the endothelium protrude into the endosperm and the ontogeny is different from Calceolariaceae, Gesneriaceae and Linderniaceae [44,106]. With regard to chemical compounds, Plocospermataceae, Oleaceae and Carlemanniaceae have no anthraquinones from the shikimic acid metabolism, Tetrachondraceae have not been examined for the occurrence of these compounds, and all other lineages in Lamiales possess them. Consequently, these anthraquinones have evolved immediately before or immediately after branching of Tetrachondraceae. Group II decarboxylated iridoids most likely evolved once after the branching of Calceolariaceae + Gesneriaceae, since they are shared by all taxa branching after this clade [1]. The close relationship between *Rehmannia *and Orobanchaceae is supported by the shared occurrence of alveolated seeds.

### Divergence ages in Lamiales

There have been several attempts to estimate Asterid divergence ages, using fossil calibration points outside Lamiales. By means of the earliest relaxed clock dating method NPRS [107], Wikström et al. [108] provided estimates for Lamiales stem group (sga) and crown group ages (cga) of 74 mya and 64 mya, respectively. Using a more sophisticated approach (PL, [107]), the later results of Bremer at al. [109] and Janssens et al. [110] were quite congruent, estimating the stem group age at 106 and 104 mya, and the crown group age at 97 and 95 mya, respectively. The recent study of Magallon and Castillo [111] presents a diversification hypothesis for all angiosperms derived from constraining minimal ages of 49 nodes with fossil data. This setup resulted in a sga of 80 mya and a cga of 63 mya for Lamiales, maybe because of the strongly reduced taxon sampling among Lamiales compared to Bremer et al. [109]. Furthermore, the highest diversification rates among angiosperms were found in Lamiales [112]. This rapid radiation could be a reason for the difficulty in untangling the relationships in Lamiales, as previously supposed [2]. The very short branches among the representatives of Higher Core Lamiales (see Figure 3) are putatively indicative of a rapid radiation. So far, reliable relaxed-clock estimates for the age of major Lamiales lineages have been lacking for two reasons, one of which is the scantiness of useful fossil calibration points. Only few fossils, sometimes with questionable assignment [113], are known from Lamiales. They include a mummified *Byblis *seed (middle Eocene[114]), a fruit from Bignoniaceae (middle Eocene, [115]), *Justicia*-like pollen (Neogene, [116]), and vegetative parts from *Hippuris *(Hippuridaceae), *Fraxinus *(Oleaceae), and *Chilopsis *(Bignoniaceae) from Oligocene [117]. The second reason for the absence of dating attempts in Lamiales has been the uncertainty with respect to the phylogenetic position of the families within Lamiales. We believe that our study represents good progress with regard to this second problem. Nevertheless, we refrain from trying to obtain divergence age estimated based on our data at this point, because (i) the sparseness of reliable and useful fossil calibration points would force us to either use an insufficient number of calibration points or use calibration points that themselves are molecular-clock based estimates with a substantial error margin, and (ii) because the remaining uncertainties in the branching order within Lamiales would translate into inferring clade ages with unsatisfyingly wide confidence intervals.

## Conclusions

### Utility of chloroplast markers for Lamiales phylogenetics

Phylogenetic analysis of combined *trnK*/*matK*, *trn*L-F and *rps*16 intron sequences enhanced both resolution and statistical support compared to previous studies. Addition of the more slowly evolving protein coding *rbcL *and *ndhF *genes to our three-marker dataset did not increase resolution and support values of trees to the slightest degree (Additional file 6, Figure S5), and analyses of each of the coding markers alone yield highly unresolved topologies.

Despite the step forward reported here, more data need to be compiled to clarify the affinities within the derived Lamiales, especially for finding the next relatives of carnivorous lineages and a better understanding of the path to carnivory in the order. A recent simulation study argued for accumulating many more characters from slow evolving markers, and recommends 10,000-20,000 characters for Lamiales [40]. Apart from the much greater effort required by this strategy, the simulation approach taken by the authors does not allow a rejection of the utility of non coding markers. This is because the distribution of rates and homoplasy at individual sites, which seems to be a very important factor determining phylogenetic utility [57], was not taken into account by the authors. Moreover, simulations were exclusively based on substitutional patterns derived from functionally highly constrained *ndhF *and *rbcL *data sets with a scarce taxon sampling and a very rough estimation of phylogeny by neighbor-joining. A currently popular approach in large scale angiosperm phylogenetics takes this idea one step further and uses concatenated coding sequences extracted from complete cp genome sequences (e.g. [118]).

However, regardless of the markers and number of characters used, it has emerged as highly crucial to maintain a high taxon sampling density while accumulating more characters [40,112,119]. Although the cost for complete cp genome sequences have dropped dramatically in the past years, in particular when only protein coding regions are targeted and no assembly is aimed at, the cost/benefit ratio so far has prevented researchers from taking this avenue for resolving the Lamiales phylogeny. For such an approach, it is currently unclear whether an appropriate number of taxa could be upheld while keeping costs at a reasonable level, and whether the information content in even a large number of slowly evolving protein coding genes would significantly exceed that in just a few more quickly evolving cp genome regions. In view of the substantial progress made here with this kind of marker, adding further data from non-protein coding chloroplast regions seems a promising strategy that, alone or in combination with phylogenomic approaches, might finally provide us with a clear picture of Lamiales evolution.

## Authors' contributions

B.S. generated data and drafted the manuscript. K.F.M. was responsible for the conception of the study and helped writing the manuscript. D.C.A. provided data and improved the manuscript. A.F. and T.B. provided plant material. T.B. contributed during manuscript preparation. A.F., E.F. and G.H. improved the manuscript. T.B., E.F., and D.C.A. contributed to the conception of the study during its initial phase, G.H. in its final phase. A.F. contributed during manuscript preparation. All authors have given final approval of the version to be published.

## Supplementary Material

Additional file 1 **Table S1: Taxa, specimens and GenBank acession numbers for sequences used in the 5 gene analysis**. Voucher information. Click here for file

Additional file 2 **Figure S1: A comparison of decay values**. Numbers above branches give decay values from nucleotide data matrix; numbers below branches that from nucleotides plus coded indels. Click here for file

Additional file 3 **Figure S2: Tree from *rbcL *analysis**. Strict consensus of 100 MP bootstrap replicates performed. Click here for file

Additional file 4 **Figure S3: Tree from *ndhF *analysis**. Strict consensus of 100 MP bootstrap replicates performed. Click here for file

Additional file 5 **Figure S4: Tree from angiosperm-wide *matK *analysis of the Hilu et al. 2003 dataset plus our newly generated *Hydrostachys *sequence**. Strict consensus of 100 MP bootstrap replicates performed. Click here for file

Additional file 6 **Figure S5: Tree from combined *trnK/matK, trnL-F, rps16, rbcL, ndhF *analysis**. 100 bootstrap replicates performed. Click here for file

## References

[B1] Stevens PF Angiosperm Phylogeny Website 2001 http://www.mobot.org/MOBOT/research/APweb/Version 7, May 2006

[B2] Rahmanzadeh R Müller KF Fischer E Bartels D Borsch T Linderniaceae and Gratiolaceae (Lamiales) are further lineages distinct from Scrophulariaceae Plant Biology 2005 7 67 78 10.1055/s-2004-830444 15666207

[B3] Bartels D Desiccation tolerance studied in the resurrection plant *Craterostigma plantagineum* Integr Comp Biol 2005 45 696 701 10.1093/icb/45.5.696 21676819

[B4] Young ND Steiner KE dePamphilis CW The evolution of prasitism in Scrophulariaceae/Orobanchaceae: plastid gene sequences refute an evolutionary transition series Ann MO Bot Gard 1999 86 876 893 10.2307/2666173

[B5] Richies CR Parker C Parasitic plants as weeds Parasitic plants 1995 London, UK: Chapman & Hall 226 255

[B6] Lloyd FE Carnivorous plants 1942 Massachusetts: Waltham

[B7] Juniper BE Robins RJ Joel DM The carnivorous plants 1989 London: Academic press

[B8] Brugger J Rutishauser R Bau und Entwicklung landbewohnender *Utricularia*-Arten Bot Helv 1989 99 91 146

[B9] Rutishauser R Sattler R Complementarity and heuristic value of contrasting models in structural botany: 3. Case study on shoot-like "leaves" and leaf-like "shoots" in *Utricularia macrorhiza *and *U. purpurea *(Lentibulariaceae) Bot Jahrb 1989 111 121 137

[B10] Rutishauser R Isler B Developmental genetics and morphological evolution of flowering plants, especially Bladderworts (*Utricularia*): Fuzzy arberian morphology complements classical morphology Ann Bot 2001 88 1173 1202 10.1006/anbo.2001.1498

[B11] Barthlott W Porembski S Fischer E Gemmel B First protozoa-trapping plant found Nature 1998 392 447 10.1038/330379548248

[B12] Greilhuber J Borsch T Müller KF Worberg A Porembski S Barthlott W Smallest angiosperm genomes found in Lentibulariaceae, with chromosomes of bacterial size Plant Biology 2006 8 770 777 10.1055/s-2006-924101 17203433

[B13] Müller KF Borsch T Legendre L Porembski S Theisen I Barthlott W Evolution of carnivory in Lentibulariaceae and the Lamiales Plant Biology 2004 6 477 490 10.1055/s-2004-817909 15248131

[B14] Müller KF Borsch T Legendre L Porembski S Barthlott W Recent progress in understanding the evolution of Lentibulariaceae Plant Biology 2006 8 748 757 10.1055/s-2006-924706 17203430

[B15] Hartmeyer S Carnivory of *Byblis *revisited-A simple method for enzyme testing on carnivorous plants Carniv Pl Newslett 1997 26 34 38

[B16] Plachno BJ Adamec L Lichtscheidl IK Peroutka M Adlassnig W Vrba J Fluorescence labelling of phosphatase activity in digestive glands of carnivorous plants Plant Biology 2006 8 813 820 10.1055/s-2006-924177 16865659

[B17] Lang F Untersuchungen über Morphologie, Anatomie und Samenentwicklung von *Polypompholyx *und *Byblis gigantea* Flora 1901 149 206

[B18] Conran JG The embryology and relationships of the Byblidaceae Aust Sys Bot 1996 9 243 254 10.1071/SB9960243

[B19] Albert VA Williams SE Chase MW Carnivorous plants: Phylogeny and structural evolution Science 1992 257 1491 1495 10.1126/science.1523408 1523408

[B20] Taylor P Souza VC Giulietti AM Harley RM *Philcoxia*: A new genus of Scrophulariaceae with three new species from Eastern Brazil Kew Bulletin 2000 55 155 163 10.2307/4117770

[B21] Fritsch P Almeda F Martins AB Cruz BC Estes D Rediscovery and phylogenetic placement of *Philcoxia minensis *(Plantaginaceae), with a test of carnivory Proc Calif Acad Sci 2007 58 447 467

[B22] Albach DC Meudt HM Oxelman B Piecing together the "new" Plantaginaceae Am J Bot 2005 92 297 315 10.3732/ajb.92.2.297 21652407

[B23] McDade LA Moody ML Phylogenetic relationships among Acanthaceae: evidence from noncoding trnL-trnF chloroplast DNA sequences Am J Bot 1999 86 70 80 10.2307/2656956 21680347

[B24] Olmstead RG Bremer B Scott KM Palmer JD A parsimony analysis of the Asteridae s.l. based on rbcL sequences Ann MO Bot Gard 1993 80 700 722 10.2307/2399855

[B25] Olmstead RG Kim KJ Jansen RK Wagstaff SJ The phylogeny of the Asteridae sensu lato based on chloroplast ndhF gene sequences Mol Phylogenet Evol 2000 16 96 112 10.1006/mpev.1999.0769 10877943

[B26] Bremer B Bremer K Heidari N Erixon P Olmstead RG Anderberg AA Källersjö M Barkhordarian E Phylogenetics of asterids based on 3 coding and 3 non-coding chloroplast DNA markers and the utility of non-coding DNA at higher taxonomic levels Mol Phylogenet Evol 2002 24 274 301 10.1016/S1055-7903(02)00240-3 12144762

[B27] Soltis DE Soltis PS Endress PK Chase MW Phylogeny and evolution of angiosperms 2005 Sunderland, Massachusetts, USA: Sinauer Associates

[B28] APG III An update of the Angiosperm Phylogeny Group classification for the orders and families of flowering plants: APG III Bot J Linn Soc 2009 161 105 121 10.1111/j.1095-8339.2009.00996.x

[B29] Dahlgren G (ed) Systematische Botanik 1987 Berlin Heidelberg New York: Springer

[B30] Takhtajan A Diversity and Classification of Flowering Plants 1997 New York: Columbia University Press

[B31] Burleigh JG Hilu K Soltis D Inferring phylogenies with incomplete data sets: a 5-gene, 567-taxon analysis of angiosperms BMC Evol Biol 2009 9 61 10.1186/1471-2148-9-61 19292928 PMC2674047

[B32] Xiang Q Moody ML Soltis DE Fan CZ Soltis PS Relationships within Cornales and circumscription of Cornaceae--matK and rbcL sequence data and effects of outgroups and long branches Mol Phylogenet Evol 2002 24 35 57 10.1016/S1055-7903(02)00267-1 12128027

[B33] Fan C Xiang Q Phylogenetic analyses of Cornales based on 26S rRNA and combined 26S rDNA-matK-rbcL sequence data Am J Bot 2003 90 1357 1372 10.3732/ajb.90.9.1357 21659236

[B34] Hempel A Reeves PA Olmstead R Jansen RK Implications of rbcL sequence data for higher order relationships of the Loasaceae and the anomalous aquatic plant *Hydrostachys *(Hydrostachyaceae) Plant Syst Evol 1994 194 25 37 10.1007/BF00983214

[B35] Hilu KW Borsch T Müller KF Soltis DE Soltis PS Savolainen V Chase M Powell M Alice LA Evans R Sauquet H Neinhuis C Slotta TA Rohwer JG Campbell CS Chatrou L Angiosperm phylogeny based on matK sequence information Am J Bot 2003 90 1758 1776 10.3732/ajb.90.12.1758 21653353

[B36] Savolainen V Fay MF Albach DC Backlund A Van der Bank M Cameron KM Johnson LA Lledó MD Pintaud J-Powell M Sheaham MC Soltis DE Soltis PS Weston P Whitten WM Wurdack KJ Chase MW Phylogeny of the eudicots: a nearly complete familial analysis based on rbcL gene sequences Kew Bulletin 2000 55 257 309 10.2307/4115644

[B37] Tank DC Beardsley PM Kelchner SA Olmstead RG L. A. S. JOHNSON REVIEW No. 7. Review of the systematics of Scrophulariaceae s.l. and their current disposition Aust J Bot 2006 19 289 307 10.1071/SB05009

[B38] Olmstead RG DePamphilis CW Wolfe AD Young ND Elisons WJ Reeves PA Disintegration of the Scrophulariaceae Am J Bot 2001 88 348 361 10.2307/2657024 11222255

[B39] Oxelman B Kornhall P Olmstead RG Bremer B Further disintegration of Scrophulariaceae Taxon 2005 54 411 425 10.2307/25065369

[B40] Wortley AH Rudall PJ Harris DJ Scotland RW How much data are needed to resolve a difficult phylogeny? Case study in Lamiales Syst Biol 2005 54 697 709 10.1080/10635150500221028 16195214

[B41] Andersson S On the phylogeny of the genus *Calceolaria *(Calceolariaceae) as inferred from ITS and plastid matK sequences Taxon 2006 55 125 137 10.2307/25065534

[B42] Wortley AH Harris DJ Scotland RW On the Taxonomy and Phylogenetic Position of *Thomandersia* Syst Botany 2007 32 415 444 10.1600/036364407781179716

[B43] Xia Z Wang Y Smith JF Familial placement and relations of *Rehmannia *and *Triaenophora *(Scrophulariaceae s.l.) inferred from five gene regions Am J Bot 2009 96 519 530 10.3732/ajb.0800195 21628207

[B44] Fischer E Kubitzki K Scrophulariaceae The Families and Genera of Vascular Plants 2004 Berlin: Springer 333 432

[B45] Olmstead RG Reeves PA Evidence for the polyphyly of the Scrophulariaceae based on chloroplast rbcL and ndhF sequences Ann MO Bot Gard 1995 82 176 193 10.2307/2399876

[B46] APG2 An update of the Angiosperm Phylogeny Group classification for the orders and families of flowering plants: APG II Bot J Linn Soc 2003 141 399 436 10.1046/j.1095-8339.2003.t01-1-00158.x

[B47] dePamphilis CW Young ND Wolfe AD Evolution of plastid gene rps2 in a lineage of hemiparasitic and holoparasitic plants: many losses of photosynthesis and complex patterns of rate variation Proc Natl Acad Sci USA 1997 94 7367 7372 10.1073/pnas.94.14.7367 9207097 PMC23827

[B48] Wolfe A dePamphilis C The effect of relaxed functional constraints on the photosynthetic gene rbcL in photosynthetic and nonphotosynthetic parasitic plants Mol Biol Evol 1998 15 1243 1258 9787431 10.1093/oxfordjournals.molbev.a025853

[B49] Bennett JR Mathews S Phylogeny of the parasitic plant family Orobanchaceae inferred from phytochrome A Am J Bot 2006 93 1039 1051 10.3732/ajb.93.7.1039 21642169

[B50] Albach DC Yan K Jensen SR Li H Phylogenetic placement of *Triaenophora *(formerly Scrophulariaceae) with some implications for the phylogeny of Lamiales Taxon 2009 58 749 756

[B51] Beardsley PM Olmstead RG Redefining Phrymaceae: The placement of *Mimulus*, tribe Mimuleae, and *Phryma* Am J Bot 2002 89 1093 1102 10.3732/ajb.89.7.1093 21665709

[B52] Magin N Classen R Gack C The morphology of false anthers in *Craterostigma plantagineum *and *Torenia polygonioides *(Scrophulariaceae) Can J Bot 1989 67 1931 1937 10.1139/b89-245

[B53] Fischer E Systematik der afrikanischen Lindernieae (Scrophulariaceae) Trop Subtrop Pflanzenwelt 1992 82 1 365

[B54] Spangler RE Olmstead RG Phylogenetic analysis of Bignoniaceae based on the cpDNA gene sequences rbcL and ndhF Ann MO Bot Gard 1999 86 33 46 10.2307/2666216

[B55] Nakai T Classes, Ordines, Familiae, Subfamiliae, Tribus, Genera nov quae attinet ad plantas Koreanas J Jap Bot 1949 24 8 14

[B56] Reveal J Newly required suprageneric names in vascular plants Phytologia 1995 79 68 76

[B57] Müller KF Borsch T Hilu KW Phylogenetic utility of rapidly evolving DNA at high taxonomical levels: Contrasting matK, trnT-F, and rbcL in basal angiosperms Mol Phylogenet Evol 2006 41 99 117 10.1016/j.ympev.2006.06.017 16904914

[B58] Borsch T Hilu KW Quandt D Wilde V Neinhuis C Barthlott W Non-coding plastid trnT-trnF sequences reveal a well resolved phylogeny of basal angiosperms J Evol Biol 2003 16 558 576 10.1046/j.1420-9101.2003.00577.x 14632220

[B59] Worberg A Quandt D Barniske A-Löhne C Hilu KW Borsch T Phylogeny of basal eudicots: Insights from non-coding and rapidly evolving DNA Org Divers Evol 2007 7 55 77 10.1016/j.ode.2006.08.001

[B60] Borsch T Quandt D Mutational dynamics and phylogenetic utility of noncoding chloroplast DNA Plant Syst Evol 2009 282 169 199 10.1007/s00606-009-0210-8

[B61] Altschul SF Gish W Miller W Myers EW Lipman DJ Basic local alignment search tool J Mol Biol 1990 215 403 410 2231712 10.1016/S0022-2836(05)80360-2

[B62] Hufford L Moody ML Soltis DE A phylogenetic analysis of Hydrangeaceae based on sequences of the plastid gene matk and their combination with rbcl and morphological data Int J Plant Sci 2001 162 835 846 10.1086/320789

[B63] Albach DC Soltis PS Soltis DE Olmstead RG Phylogenetic analysis of asterids based on sequences of four genes Ann MO Bot Gard 2001 88 163 212 10.2307/2666224

[B64] Müller J Müller KF Neinhuis C Quandt D PhyDE - Phylogenetic Data Editor 2006 http://www.phyde.de

[B65] Kelchner SA The evolution of non-coding chloroplast DNA and its application in plant systematics Ann MO Bot Gard 2000 87 482 498 10.2307/2666142

[B66] Simmons MP Ochoterena H Gaps as characters in sequence-based phylogenetic analyses Syst Biol 2000 49 369 381 10.1093/sysbio/49.2.369 12118412

[B67] Müller KF SeqState: Primer design and sequence statistics for phylogenetic DNA datasets Appl Bioinformatics 2005 4 65 69 10.2165/00822942-200504010-00008 16000015

[B68] Müller KF PRAP - computation of Bremer support for large data sets Mol Phylogenet Evol 2004 31 780 782 10.1016/j.ympev.2003.12.006 15062810

[B69] Swofford DL PAUP*. Phylogenetic Analysis Using Parsimony (* and other Methods) 1998 Sinauer Associates, Sunderland, Massachussets

[B70] Müller KF The efficiency of different search strategies in estimating parsimony jackknife, bootstrap, and Bremer support BMC Evol Biol 2005 5 58 10.1186/1471-2148-5-58 16255783 PMC1282575

[B71] Ronquist F Huelsenbeck JP MrBayes 3: Bayesian phylogenetic inference under mixed models Bioinformatics 2003 19 1572 1574 10.1093/bioinformatics/btg180 12912839

[B72] Posada D jModelTest: phylogenetic model averaging Mol Biol Evol 2008 25 1253 1256 10.1093/molbev/msn083 18397919

[B73] Andrew Rambaut Alexei Drummond J Tracer 2009 Edinburgh: Institute for Evolutionary Biology

[B74] Stamatakis A RAxML-VI-HPC: maximum likelihood-based phylogenetic analyses with thousands of taxa and mixed models Bioinformatics 2006 22 2688 2690 10.1093/bioinformatics/btl446 16928733

[B75] Stöver B Müller KF TreeGraph 2: Combining and visualizing evidence from different phylogenetic analyses BMC Bioinformatics 2010 11 7 10.1186/1471-2105-11-7 20051126 PMC2806359

[B76] Shimodaira H An approximately unbiased test of phylogenetic tree selection Syst Biol 2002 51 492 508 10.1080/10635150290069913 12079646

[B77] Shimodaira H Hasegawa M Multiple comparisons of log-likelihoods with applications to phylogenetic inference Mol Biol Evol 1999 16 1114 1116

[B78] Shimodaira H Hasegawa M CONSEL: for assessing the confidence of phylogenetic tree selection Bioinformatics 2001 17 1246 1247 10.1093/bioinformatics/17.12.1246 11751242

[B79] Watson L Dallwitz M The families of flowering plants: descriptions, illustrations, identification, and information retrieval 1992

[B80] Mabberley D The plant-book: a portable dictionary of the vascular plants 2008 Cambridge: Cambridge University Press

[B81] Pagel M Meade A Barker D Bayesian Estimation of Ancestral Character States on Phylogenies Systematic Biology 2004 53 673 684 10.1080/10635150490522232 15545248

[B82] Hufford L McMahon MM Sherwood AM Reeves G Chase MW The major clades of Loasaceae: Phylogenetic analysis using the plastid matK and trnL-trnF regions Am J Bot 2003 90 1215 1228 10.3732/ajb.90.8.1215 21659222

[B83] Jäger-Zürn I Zur Frage der systematischen Stellung der Hydrostachyaceae auf Grund ihrer Embryologie, Blüten-und Infloreszenzmorphologie Plant Syst Evol 1965 112 621 639 10.1007/BF01373191

[B84] Soltis DE Soltis PS Chase MW Mort ME Albsch DC Zanis M Savolainen V Hahn WH Hoot SB Fay MF Axtell M Swensen SM Prince LM Kress WJ Nixon KC Farris JS Angiosperm phylogeny inferred from 18S rDNA, rbcL, and atpB sequences Bot J Linn Soc 2000 133 81 381-461

[B85] Albach D Phylogenetic placement of the enigmatic angiosperm *Hydrostachys* Taxon 2001 50 781 805 10.2307/1223707

[B86] Jensen SR Albach DC Ohno T Grayer RJ *Veronica*: Iridoids and cornoside as chemosystematic markers Biochem Syst Ecol 2005 33 1031 1047 10.1016/j.bse.2005.03.001

[B87] Smith JF Wolfram JC Brown KD Carroll CL Denton DS Tribal Relationships in the Gesneriaceae: evidence from DNA sequences of the chloroplast gene ndhF Ann MO Bot Gard 1997 84 50 66 10.2307/2399953

[B88] Bentham G Bentham G, Hooker JD Gesneriaceae Genera Plantarum 2 1876 990 1025 Reeve

[B89] Estes D Small RL Phylogenetic relationships of the monotypic genus *Amphianthus *(Plantaginaceae tribe Gratioleae) inferred from chloroplast DNA sequences Syst Botany 2008 33 176 182 10.1600/036364408783887375

[B90] Soltis DE Soltis PS Endress PK Chase MW Phylogeny and Evolution of Angiosperms 2005

[B91] Felsenstein J Cases in which parsimony or compatibility methods will be positively misleading Syst Biol 1978 27 401 410

[B92] McDade LA Daniel TF Kiel CA Toward a comprehensive understanding of phylogenetic relationships among lineages of Acanthaceae s.l. (Lamiales) Am J Bot 2008 95 1136 1152 10.3732/ajb.0800096 21632432

[B93] Plachno BJ Adamec L Huet H Mineral nutrient uptake from prey and glandular phosphatase activity as a dual test of carnivory in semi-desert plants with glandular leaves suspected of carnivory Ann Bot-London 2009 104 649 654 10.1093/aob/mcp155 PMC2729641 19556266

[B94] Rice B Reassessing commensal-enabled carnivory in *Proboscidea *and *Ibicella*? Carniv Pl Newslett 2008 15 19

[B95] Bremer B Olmstead RG Struwe L Sweere JA rbcL sequences support exclusion of *Retzia*, *Desfontainia*, and *Nicodemia *from the Gentianales Plant Syst Evol 1994 190 213 230 10.1007/BF00986194

[B96] Oxelman B Backlund M Bremer B Relationships of the Buddlejaceae s.l. investigated using parsimony jackknife and branch support analysis of chloroplast ndhF and rbcL sequence data Syst Botany 1999 24 164 182 10.2307/2419547

[B97] Cronquist A The Evolution and Classification of Flowering Plants 1988 2 New York: The New York Botanical Garden

[B98] Briquet J Engler A, Prantl K Labiatae Die natürlichen Pflanzenfamilien 1895 4/3a Leipzig: Engelmann 132 182

[B99] Cantino PD Evidence for a polyphyletic origin of the Labiatae Ann MO Bot Gard 1992 361 379 10.2307/2399774

[B100] Wagstaff SJ Hickerson L Spangler R Reeves PA Olmstead RG Phylogeny in Labiatae s.l., inferred from cpDNA sequences Plant Syst Evol 1998 209 265 274 10.1007/BF00985232

[B101] Wortley AH Scotland RW Rudall PJ Floral anatomy of *Thomandersia *(Lamiales), with particular reference to the nature of the retinaculum and extranuptial nectaries Bot J Linn Soc 2005 149 469 10.1111/j.1095-8339.2005.00507.x

[B102] Mayr EM Weber A Calceolariaceae: floral development and systematic implications Am J Bot 2006 93 327 343 10.3732/ajb.93.3.327 21646194

[B103] Bello MA Rudall PJ González F Fernández-Alonso JL Floral morphology and development in *Aragoa *(Plantaginaceae) and related members of the order Lamiales Int J Plant Sci 2004 165 723 738 10.1086/422046

[B104] Albach DC Martinez-Ortega MM Fischer MA Chase MW Evolution of Veroniceae: A phylogenetic perspective Ann MO Bot Gard 2004 91 275 302

[B105] Endress P Symmetry in flowers: diversity and evolution Int J Plant Sci 1999 160 S3 S23 10.1086/314211 10572019

[B106] Hartl D Das alveolierte Endosperm bei Scrophulariaceen, seine Entstehung, Anatomie und taxonomische Bedeutung Beiträge zur Biologie der Pflanzen 1959 35 95 110

[B107] Sanderson MJ Estimating absolute rates of molecular evolution and divergence times: a penalized likelihood approach Mol Biol Evol 2002 19 101 109 11752195 10.1093/oxfordjournals.molbev.a003974

[B108] Wikström N Savolainen V Chase MW Evolution of the angiosperms: calibrating the family tree Proc R Soc Lond [Biol] 2001 268 2211 2220 10.1098/rspb.2001.1782 PMC1088868 11674868

[B109] Bremer K Friis EM Bremer B Molecular phylogenetic dating of asterid flowering plants shows early Cretaceous diversification Syst Biol 2004 53 496 505 10.1080/10635150490445913 15503676

[B110] Janssens SB Knox EB Huysmans S Smets EF Merckx VS Rapid radiation of *Impatiens *(Balsaminaceae) during Pliocene and Pleistocene: Result of a global climate change Mol Phylogenet Evol 2009 52 806 824 10.1016/j.ympev.2009.04.013 19398024

[B111] Magallon S Castillo A Angiosperm diversification through time Am J Bot 2009 96 349 365 10.3732/ajb.0800060 21628193

[B112] Zwickl DJ Hillis DM Increased taxon sampling greatly reduces phylogenetic error Syst Biol 2002 51 588 598 10.1080/10635150290102339 12228001

[B113] Nie Z Sun H Beardsley PM Olmstead RG Wen J Evolution of biogeographic disjunction between eastern Asia and eastern North America in *Phryma *(Phrymaceae) American Journal of Botany 2006 93 1343 1356 10.3732/ajb.93.9.1343 21642199

[B114] Conran JG Christophel DC A fossil Byblidaceae seed from Eocene South Australia Int J Plant Sci 2004 165 691 694 10.1086/386555

[B115] Pigg KB Wehr WC Tertiary Flowers, Fruits, and Seeds of Washington State and Adjacent Areas-Part III Wash Geol 2002 30 3 16

[B116] Germeraad J Hopping C Muller J Palynology of tertiary sediments from tropical areas Review of Palaeobotany and Palynology 1968 6 189 198 200-210, 212-228, 230-259, 261, 263-348 10.1016/0034-6667(68)90051-1

[B117] Axelrod DI The Late Oligocene Creede Flora, Colorado 1987 130 Berkeley, Los Angeles, London: University of California Press

[B118] Jansen RK Cai Z Raubeson LA Daniell H dePamphilis CW Leebens-Mack J Müller KF Guisinger-Bellian M Haberle RC Hansen AK Chumley TW Lee S Peery R McNeal JR Kuehl JV Boore JL Analysis of 81 genes from 64 plastid genomes resolves relationships in angiosperms and identifies genome-scale evolutionary patterns Proc Natl Acad Sci USA 2007 104 19369 19374 10.1073/pnas.0709121104 18048330 PMC2148296

[B119] Qiu Y Li L Wang B Chen Z Knoop V Groth-Malonek M Dombrovska O Lee J Kent L Rest J Estabrook GF Hendry TA Taylor DW Testa CM Ambros M Crandall-Stotler B Duff RJ Stech M Frey W Quandt D Davis CC The deepest divergences in land plants inferred from phylogenomic evidence Proc Natl Acad Sci USA 2006 103 15511 15516 10.1073/pnas.0603335103 17030812 PMC1622854

[B120] Johnson LA Soltis DE Phylogenetic inference in Saxifragaceae s.str. and *Gilia *(Polemoniaceae) using matK sequences Ann MO Bot Gard 1995 82 149 175 10.2307/2399875

[B121] Müller KF Borsch T Phylogenetics of Amaranthaceae based on matK/trnK sequence data evidence from parsimony, likelihood, and Bayesian analyses Ann MO Bot Gard 2005 92 66 102

[B122] Taberlet P Gielly L Pautou G Bouvet J Universal primers for amplification of 3 noncoding regions of chloroplast DNA Plant Mol Biol 1991 17 1105 1109 10.1007/BF00037152 1932684

[B123] Oxelman B Lidén M Berglund D Chloroplast rps16 intron phylogeny of the tribe Sileneae (Caryophyllaceae) Plant Syst Evol 1997 206 393 410 10.1007/BF00987959

