# Supplementary material for: Towards resolving Lamiales relationships: insights from rapidly evolving chloroplast sequences
Source: BMC Evol Biol. 2010 Nov 12;10:352. doi: 10.1186/1471-2148-10-352 (PMC2992528; doi:10.1186/1471-2148-10-352)
Supplement: Additional file 1 — Table S1: Taxa, specimens and GenBank acession numbers for sequences used in the 5 gene analysis. Voucher information. [file 1471-2148-10-352-S1.DOC]

|  | Family | *rbcL* | *ndhF* |
| --- | --- | --- | --- |
| *Acanthus* | Acanthaceae | *Acanthus montanus* T.Anderson; L12592.1 | *Acanthus montanus* T.Anderson; AJ429115.1 |
| *Anastrabe* | Stilbaceae | - | *Anastrabe integerrima* E. Mey. Ex Benth.; AJ619551.1 |
| *Angelonia* | Gratiolaceae | *Angelonia pubescens* Benth.; AF123672.1 | *Angelonia angustifolia* Benth.; AJ617588.1 |
| *Antirrhinum* | Plantaginaceae | *Antirrhinum majus* L.; L11688.1 | *Antirrhinum majus* L.; L36392.1 |
| *Avicennia* | Acanthaceae | *Avicennia germinans* L.; AY008830.1 | *Avicennia* *marina* (Forssk.) Vierh.; AJ429116.1 |
| *Bacopa* | Gratiolaceae | *Bacopa caroliniana* B.L.Rob.; AF123670.1 | *Bacopa monnieri* (L.) Pennell; EF527447.1 |
| *Barthlottia* | Scrophulariaceae | - | *Barthlottia madagascariensis* Eb.Fisch.; AJ401438.1 |
| *Buchnera* | Orobanchaceae | *Buchnera floridana* Gand.; AF026822.1 | - |
| *Buddleja* | Scrophulariaceae | *Buddleja davidii* Franch.; AJ001757.1 | *Buddleja davidii* Franch.; AF130143.1 |
| *Byblis* | Byblidaceae | *Byblis liniflora* Salisb.; AB546625.1 | - |
| *Calceolaria* | Calceolariaceae | *Calceolaria sp*.; AF123669.1 | *Calceolaria sp*.; AF123679.1 |
| *Callicarpa* | Lamiaceae | *Callicarpa dichotoma* Raeusch.; L14393.1 | *Callicarpa mollis* Siebold & Zucc.; AY310134.1 |
| *Campsis* | Bignoniaceae | *Campsis radicans* Seem.; AF190428.1 | *Campsis* *radicans* Seem.; AF102626.1 |
| *Carlemannia* | Carlemanniaceae | *Carlemannia tetragona* Hook.f.; DQ673316.1 | *Carlemannia tetragona* Hook.f.; DQ673290.1 |
| *Castilleja* | Orobanchaceae | *Castilleja* *linariifolia* Benth.; AF026823.1 | - |
| *Clerodendrum* | Lamiaceae | *Clerodendrum inerme* (L) Gaertn.; AY289684.1 | *Clerodendrum thomsoniae* Balf.; AY115887.1 |
| *Dermatobotrys* | Scrophulariaceae | - | *Dermatobotrys* *saundersii* Bolus; AJ617592.1 |
| *Diascia* | Scrophulariaceae | *Diascia longicornis* Druce; AM235134.1 | *Diascia capsularis* Benth.; AJ617593.1 |
| *Diclis* | Scrophulariaceae | *Diclis reptans* Benth.; AM235135.1 | *Diclis reptans* Benth.; AJ619557.1 |
| *Dipteracanthus* | Acanthaceae | *Ruellia graecizans* Backer; L12595.1 | *Ruellia ciliosa* Pursh; RCU12664 |
| *Elytraria* | Acanthaceae | *Elytraria crenata* Vahl; AF188127.1 | *Elytraria crenata* Vahl; ECU12657 |
| *Euphrasia* | Orobanchaceae | *Euphrasia spectabilis* Phil.; AY849864.1 | - |
| *Forsythia* | Oleaceae | - | *Forsythia europaea* Degen & Baldacci; DQ673264.1 |
| *Genlisea* | Lentibulariaceae | *Genlisea guianensis* N.E.Br.; AY128631.1 | - |
| *Globularia* | Plantaginaceae | *Globularia cordifolia* L.; AF124558.1 | *Globularia cordifolia* L.; AF124557.1 |
| *Gratiola* | Gratiolaceae | *Gratiola neglecta* Torr.; DQ006125.1 | *Gratiola officinalis* L.; EF527461.1 |
| *Halleria* | Stilbaceae | *Halleria lucida* L.; AF026828.1 | *Halleria lucida* L.; AJ550569.1 |
| *Harpagophytum* | Pedaliaceae | *Harpagophytum grandidieri* Baill.; L01923.2 | - |
| *Harveya* | Orobanchaceae | *Harveya capensis* Hook.; DQ017800.1 | - |
| *Hydrotriche* | Gratiolaceae | *Hydrotriche hottoniaeflora* Zucc.; EF467904.1 | *Hydrotriche hottoniaeflora* Zucc.; EF467909.1 |
| *Jacaranda* | Bignoniaceae | *Jacaranda sparrei* A.H.Gentry; AF102647 | *Jacaranda mimosifolia* D.Don; EF105012.1 |
| *Jasminum* | Oleaceae | *Jasminum nudiflorum* Lindl.; AF531779.1 | *Jasminum nudiflorum* Lindl.; AF531779.1 |
| *Jovellana* | Calceolariaceae | *Jovellana sp*.; AF123666.1 | *Jovellana sp*.; AF123684.1 |
| *Kigelia* | Bignoniaceae | *Kigelia africana* Benth.; AF102648.1 | *Kigelia africana* Benth.; AF102632.1 |
| *Kohleria* | Gesneriaceae | *Kohleria spicata* Oerst.; AF170236.1 | *Kohleria hirsuta* Regel; AY623159.1 |
| *Lamium* | Lamiaceae | *Lamium maculatum* L.; Z37402.1 | *Lamium purpureum* L.; LPU78694 |
| *Lantana* | Verbenaceae | *Lantana camara* L.; AF156736.1 | *Lantana horrid* Kunth; AF130152 |
| *Limnophila* | Gratiolaceae | *Limnophila sessiliflora* Blume; GU135206.1 | *Limnophila sessiliflora* Blume; EF527458.1 |
| *Limosella* | Scrophulariaceae | - | *Limosella aquatica* L.; AJ619558.1 |
| *Lindenbergia* | Orobanchaceae | *Lindenbergia philippinensis* Benth.; AF123664.1 | *Lindenbergia philippinensis* Benth.; AF123686.1 |
| *Lindernia* | Linderniaceae | *Lindernia crustacea* (L.) F.Muell.; AB259807.1 | *Lindernia dubia* (L.) Pennell; EF527446.1 |
| *Mazus* | Phrymaceae | *Mazus omeiensis* H.L.Li; FJ172731.1 | *Mazus stachydifolius* Maxim.; AJ619559.1 |
| *Mecardonia* | Gratiolaceae | - | *Mecardonia acuminata* Small; EF527449.1 |
| *Micranthemum* | Linderniaceae | - | *Micranthemum glomeratum* (Chapm.) Shinners ; AJ617603.1 |
| *Mimulus* | Phrymaceae | *Mimulus aurantiacus* Curtis; AF026835.1 | *Mimulus aurantiacus* Curtis; AF188186.1 |
| *Mitraria* | Gesneriaceae | - | *Mitraria coccinea* Cav.; MCU62193 |
| *Myoporum* | Scrophulariaceae | *Myoporum mauritianum* A.DC.; L36445.1 | *Myoporum mauritianum* A.DC.; L36403.1 |
| *Ocimum* | Lamiaceae | *Ocimum basilicum* L.; Z37425.1 | - |
| *Oftia* | Scrophulariaceae | *Oftia africana* Bocq. Ex Baill.; AM235143.1 | *Oftia africana* Bocq. Ex Baill.; AJ617606.1 |
| *Olea* | Oleaceae | *Olea_europaea* L.; AM229542.1 | *Olea_europaea* L.; AM229542.1 |
| *Orobanche* | Orobanchaceae | *Orobanche fasciculata* Nutt.; OFU73970 | - |
| *Otacanthus* | Gratiolaceae | - | *Otacanthus azureus* (Linden) Ronse; EF527468.1 |
| *Paulownia* | Paulowniaceae | *Paulownia tomentosa* (Thunb.) Steud.; L36447.1 | *Paulownia tomentosa* (Thunb.) Steud.; L36406.1 |
| *Pedicularis* | Orobanchaceae | *Pedicularis coronata* ; AF206803.1 | *Pedicularis foliosa* L.; AF123689.1 |
| *Petrea* | Verbenaceae | *Petrea racemosa* Nees; PRU28879 | *Petrea racemosa* Nees; AY919283.1 |
| *Philcoxia* | Gratiolaceae | *Philcoxia minensis* V.C.Souza & Giul.; EF467907.1 | *Philcoxia minensis* V.C.Souza & Giul.; EF467912.1 |
| *Phryma* | Phrymaceae | *Phryma leptostachya* L.; FJ172735.1 | *Phryma leptostachya* L.; AJ617586.1 |
| *Phyla* | Verbenaceae | *Phyla lanceolata* (Michx.) Greene; AF206810.1 | *Phyla incise* Small; AF130153 |
| *Pinguicula* | Lentibulariaceae | *Pinguicula agnata* Casper; AY128627 | - |
| *Pinguicula* | Lentibulariaceae | *Pinguicula ehlersiae* Speta & F.Fuchs; AF482523.1 | - |
| *Plantago* | Plantaginaceae | *Plantago lanceolata* L.; L36454.1 | *Plantago media* L.; AY818913.1 |
| *Plocosperma* | Plocospermataceae | *Plocosperma_buxifolium* Benth.; Z68829.1 | *Plocosperma_buxifolium* Benth.; AJ011985.1 |
| *Polypremum* | Tetrachondraceae | *Polypremum_procumbens* L.; AJ011989.1 | *Polypremum_procumbens* L.; AJ011986.1 |
| *Proboscidea* | Martyniaceae | *Proboscidea louisiana* (Mill.) Thell.; L01946.2 | *Proboscidea louisiana* (Mill.) Thell.; AF123690.1 |
| *Rehmannia* |  | *Rehmannia glutinosa* Steud.; FJ172725.1 | *Rehmannia chingii* H.L.Li; EF522187.1 |
| *Rhynchoglossum* | Gesneriaceae | *Rhynchoglossum notonianum* (Wall.) B.L.Burtt; AF206817.1 | *Rhynchoglossum notonianum* (Wall.) B.L.Burtt; RNU62179 |
| *Salvia* | Lamiaceae | *Salvia guaranitica* A.St.-Hil. ex Benth.; AY570407.1 | *Salvia divinorum* Epling & Játiva; SDU78703 |
| *Schlegelia* | Schlegeliaceae | *Schlegelia parviflora* (Oerst.) Monach.; L36448.1 | *Schlegelia parviflora* (Oerst.) Monach.; L36410.1 |
| *Scoparia* | Gratiolaceae | - | *Scoparia dulcis* L.; EF527450.1 |
| *Scrophularia* | Scrophulariaceae | *Scrophularia sp*.; L36449.1 | *Scrophularia canina* L.; AM503848.2 |
| *Sesamum* | Pedaliaceae | *Sesamum indicum* L.; L14408.1 | *Sesamum indicum* L.; L36413.1 |
| *Seymeria* | Orobanchaceae | *Seymeria pectinata* Pursch; AF026837.1 | *Seymeria pectinata* Pursch; AF123691.1 |
| *Stachytarpheta* | Verbenaceae | *Stachytarpheta dichotoma* Vahl; SDU32161 | *Stachytarpheta dichotoma* Vahl; L36414.1 |
| *Stemodia* | Gratiolaceae | - | *Stemodia schottii* Holz.; EF527470.1 |
| *Stemodiopsis* | Linderniaceae | - | *Stemodiopsis buchananii* Skan; AJ619570.1 |
| *Stilbe* | Stilbaceae | *Stilbe vestita* P.J.Bergius; Z68827.1 | *Stilbe albiflora* E.Mey.; AF027287 |
| *Streptocarpus* | Gesneriaceae | *Streptocarpus holstii* Engl.; L14409.1 | *Streptocarpus holstii* Engl.; L36415.1 |
| *Tetrachondra* | Tetrachondraceae | *Tetrachondra hamiltonii* Petrie; THU28885 | *Tetrachondra_patagonica* Skotsb.; AF027272 |
| *Tetranema* | Plantaginaceae | *Tetranema mexicanum* Benth.; AF236128.1 | *Tetranema mexicanum* Benth.; AF123692.1 |
| *Thomandersia* | Thomandersiaceae | *Thomandersia hensii* De Wild. Et T. Durand; AY919279.1 | *Thomandersia hensii* De Wild. Et T. Durand; AY919284.1 |
| *Thunbergia* | Acanthaceae | *Thunbergia usambarica* Lindau; L12596.1 | *Thunbergia alata* Sims; TAU12667 |
| *Torenia* | Linderniaceae | *Torenia fournieri* Linden ex Fourn.; AF026842.1 | *Torenia polygonoides* Benth.; AJ619574.1 |
| *Utricularia* | Lentibulariaceae | - | - |
| *Utricularia* | Lentibulariaceae | - | - |
| *Verbena* | Verbenaceae | *Verbena officinalis* L.; Z37473.1 | *Verbena bracteata* Lag. & Rodr.; L36418.1 |
| *Vitex* | Lamiaceae | *Vitex agnus-castus* L.; VAU78716 | - |
| *outgroups* |  |  |  |
| *Coffea* | Rubiaceae | EF044213 | EF044213 |
| *Nicotiana* | Solanaceae | NC001879.2 | NC001879.2 |
| *Solanum* | Solanaceae | DQ231562 | DQ231562 |
