# Supplementary figures and images for: Towards resolving Lamiales relationships: insights from rapidly evolving chloroplast sequences
Source: BMC Evol Biol. 2010 Nov 12;10:352. doi: 10.1186/1471-2148-10-352 (PMC2992528; doi:10.1186/1471-2148-10-352)

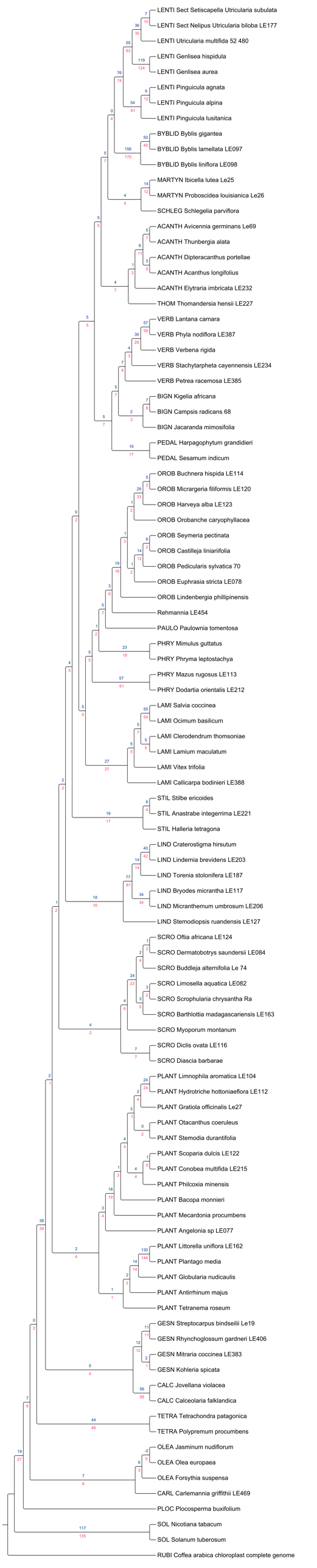

Supplement: Additional file 2 — Figure S1: A comparison of decay values. Numbers above branches give decay values from nucleotide data matrix; numbers below branches that from nucleotides plus coded indels. [file 1471-2148-10-352-S2.PDF]

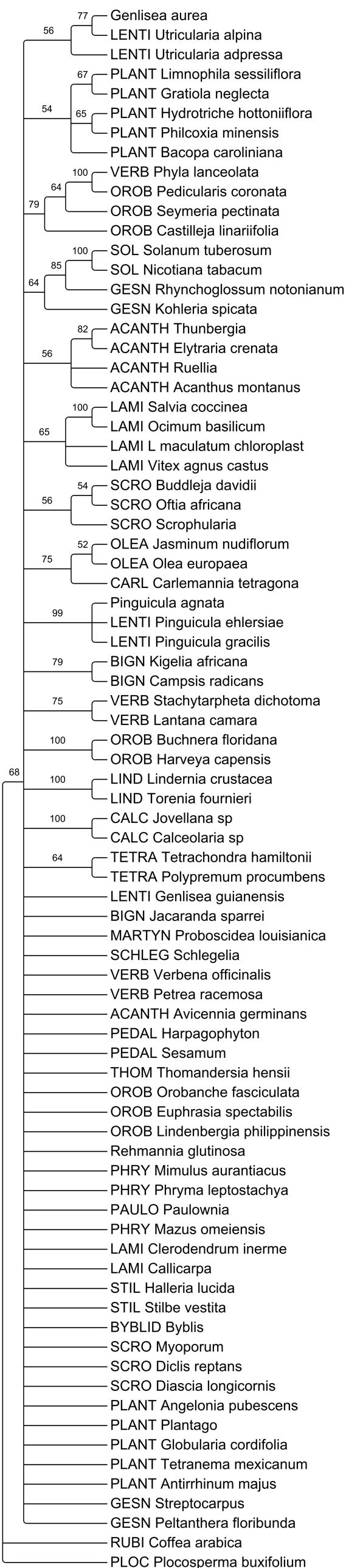

Supplement: Additional file 3 — Figure S2: Tree from rbcL analysis. Strict consensus of 100 MP bootstrap replicates performed. [file 1471-2148-10-352-S3.PDF]

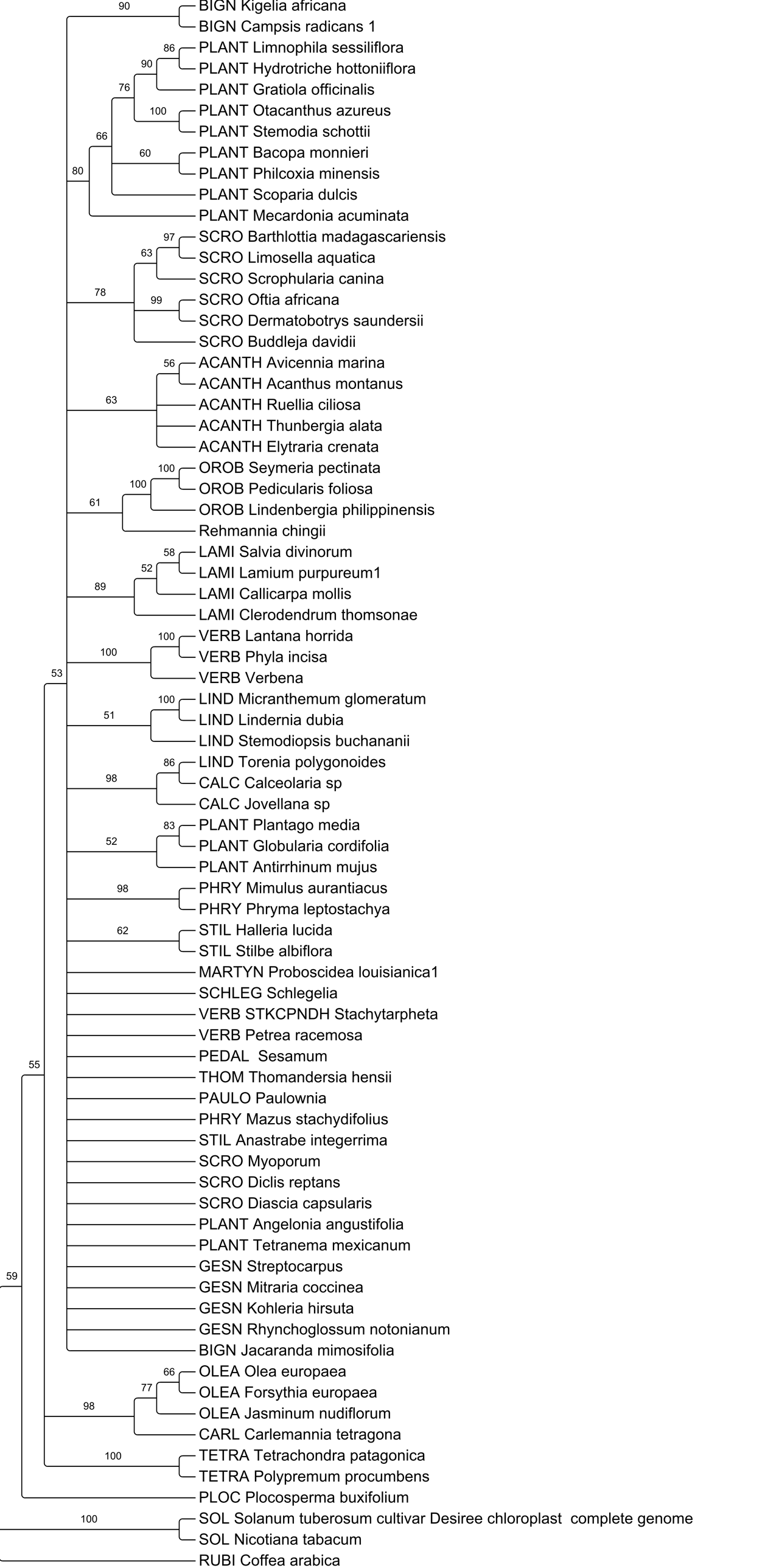

Supplement: Additional file 4 — Figure S3: Tree from ndhF analysis. Strict consensus of 100 MP bootstrap replicates performed. [file 1471-2148-10-352-S4.PDF]

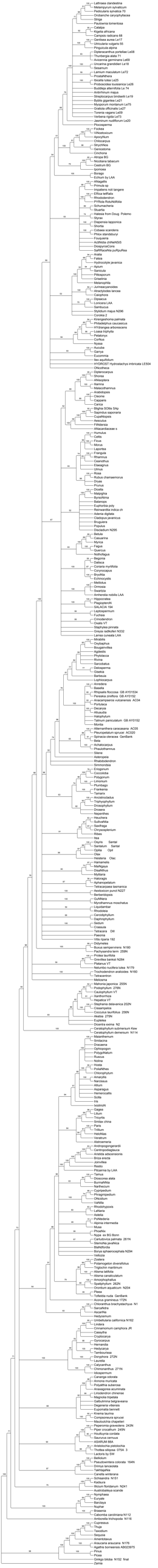

Supplement: Additional file 5 — Figure S4: Tree from angiosperm-wide matK analysis of the Hilu et al. 2003 dataset plus our newly generated Hydrostachys sequence. Strict consensus of 100 MP bootstrap replicates performed. [file 1471-2148-10-352-S5.PDF]

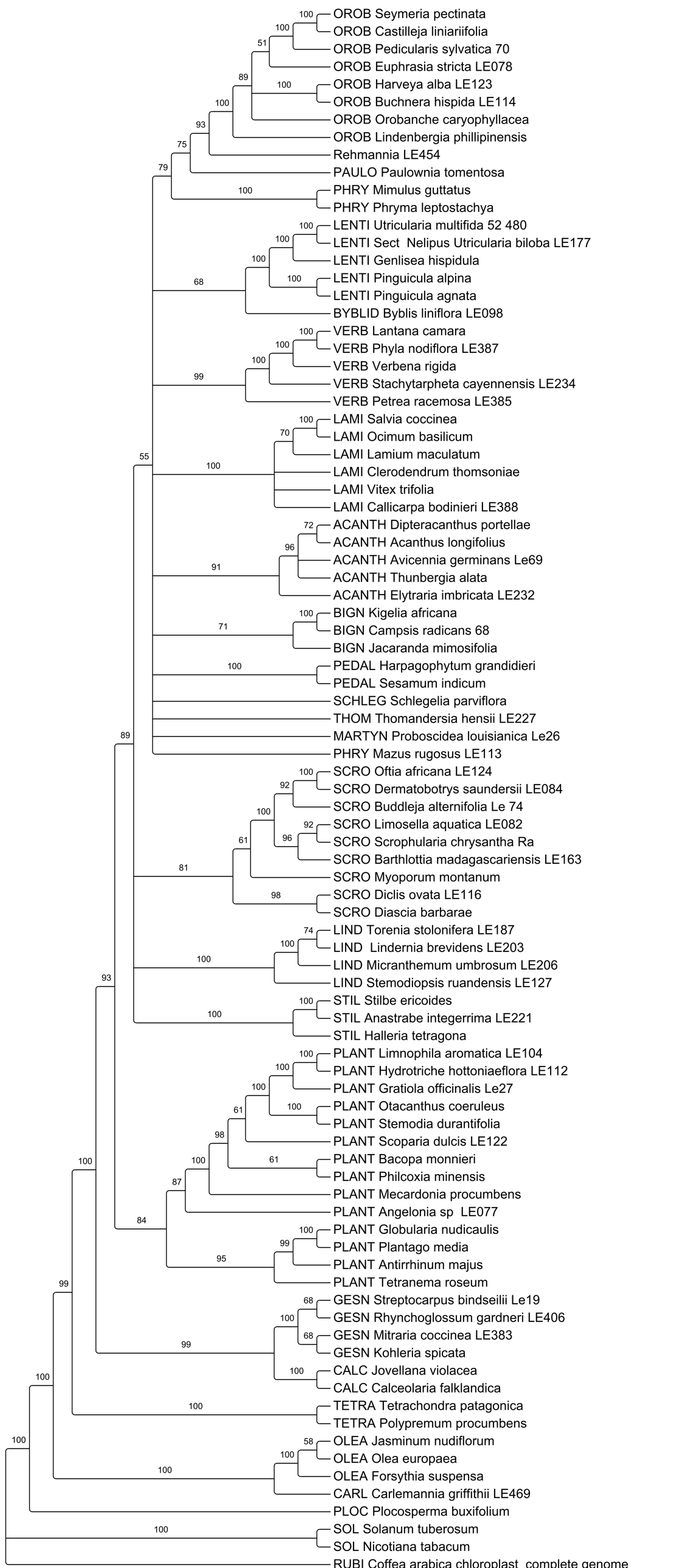

Supplement: Additional file 6 — Figure S5: Tree from combined trnK/matK, trnL-F, rps16, rbcL, ndhF analysis. 100 bootstrap replicates performed. [file 1471-2148-10-352-S6.PDF]
